# Supplementary material for: Inhibition of Chk2 promotes neuroprotection, axon regeneration, and functional recovery after CNS injury
Source: Sci Adv. 2022 Sep 14;8(37):eabq2611. doi: 10.1126/sciadv.abq2611 (PMC9473583; doi:10.1126/sciadv.abq2611)
Supplement: 20220914-1 [file sciadv.abq2611.v1.pdf]

NEUROSCIENCE

Inhibition of Chk2 promotes neuroprotection, axon regeneration, and functional recovery after CNS injury

Matthew J. Taylor[1], Adam M. Thompson[2], Sharif Alhajlah[3], Richard I. Tuxworth[1]*†, Zubair Ahmed[2,4]*†

Copyright © 2022 The Authors, some rights reserved; exclusive licensee American Association for the Advancement of Science. No claim to original U.S. Government Works. Distributed under a Creative Commons Attribution License 4.0 (CC BY).

DNA double-strand breaks occur in many acute and long-term neurological conditions, including neurodegeneration, neurotrauma, and stroke. Nonrepaired breaks chronically activate the DNA damage response in neurons, leading to neural dysfunction and apoptosis. Here, we show that targeting of the central ATM-Chk2 pathway regulating the response to double-strand breaks slows neural decline in *Drosophila* models of chronic neurodegeneration. Inhibitors of ATM-Chk2, but not the parallel ATR-Chk1 pathway, also promote marked, functional recovery after acute central nervous system injury in rats, suggesting that inhibiting nonhomologous end-joining rather than homologous recombination is crucial for neuroprotection. We demonstrate that the Chk2 inhibitor, prexasertib, which has been evaluated in phase 2 clinical trials for cancer, has potent neuroprotective effects and represents a new treatment option to promote functional recovery after spinal cord or optic nerve injury.

INTRODUCTION

The response to DNA damage and its repair is critical in the maintenance of cellular integrity, function, and survival. DNA damage may arise during normal DNA function such as replication and transcription, from endogenous sources such as reactive oxygen species (ROS), or from exogenous genotoxic agents such as mutagens and ionizing radiation (*1*). Multiple different forms of DNA damage can occur, but double-stranded breaks (DSBs) are particularly genotoxic, can cause mutation and genome instability, and, if unrepaired, lead to cell death (*2*). In replicating cells, this instability can lead to apoptosis or cellular transformation, but in the case of nonreplicating cells such as neurons, DSBs are potentially even more dangerous since the cells cannot readily be replaced.

DSBs are sensed and processed by the MRN complex, comprising Mre11, Rad50, and NBS1/Nbn proteins (*3*), which recruits ataxia telangiectasia–mutated (ATM) kinase and promotes its autophosphorylation and activation (*4*). ATM and its sister kinase ataxia telangiectasia and Rad3-related (ATR) mediate many of the downstream events such as cell cycle arrest, DNA repair, and apoptosis through activation of either checkpoint kinase-2 (Chk2) or Chk1, respectively (*5*). Thus, these kinases act as master regulators of the DNA damage response (DDR). ATM is particularly important for the response to DSBs in heterochromatin regions and regulates both nonhomologous end joining (NHEJ) and homologous recombination repair (HRR), and its signaling through Chk2 and p53 acts as a brake on the cell cycle at the G_1/S checkpoint. ATR signals through Chk1 and is generally involved in the response to replication stress where a sister chromatid is present. Hence, ATR has a more prominent role in regulating HRR in the S and G_2 phases of the cell cycle (*6*).

DSBs in DNA accumulate in many acute and chronic neurological conditions, and the failure to repair them fully causes persistent activation of the DDR. In turn, this leads to neural dysfunction, senescence, and apoptosis (*7*–*11*). We have shown recently that targeting the MRN complex to reduce persistent activation of the DDR in neurons is able to suppress the decline in neural activity in fruit fly models of chronic neurodegeneration (*12*). Moreover, the same approach provokes marked neuroprotective effects after acute central nervous system (CNS) injury in rats: Inhibiting the MRN complex prevents apoptosis of neurons, promotes axon regeneration after optic injury, and promotes axon regeneration and functional recovery after spinal cord injury (SCI) (*12*). Here, we investigated the DDR pathways regulating the neuroprotective effects in models of both chronic and acute neurological disease. We present evidence that ATM-Chk2 is the critical pathway for neuroprotection.

CNS injury is a major cause of disability among the young and represents an area of unmet clinical need. Here, we show that inhibiting Chk2 with clinically relevant small-molecule inhibitors promotes rapid, functional recovery in rats from spinal cord and optic nerve injury. Chk2, therefore, represents a new potential therapeutic target. Moreover, we highlight the dual-specificity Chk1/Chk2 inhibitor, prexasertib, which has completed phase 2 trials for cancer, that has potent neuroprotective effects at low doses and is a viable candidate to repurpose for treating CNS injuries, SCI. Hence, prexasertib would represent the first fully restorative treatment for SCI.

RESULTS

Knockdown of ATM and Chk2 is neuroprotective in *Drosophila*

Since persistent activation of the DNA damage pathway causes neuronal dysfunction and, potentially, apoptosis, then suppression of the ATM-Chk2 or ATR-Chk1 pathways may be protective to neurons and help preserve function. We tested this in an adult-onset paradigm of chronic amyloid toxicity in *Drosophila* where DSBs form in neurons (*12*, *13*) and observed a clear protective effect by knocking down expression of ATM in the $A\beta_{1-42}$-expressing adult neurons (Fig. 1A). Knockdown of Chk2, a key target downstream of ATM, in the DDR was also protective (Fig. 1B). Given that neurons are postmitotic, it is arguable that their main DSB repair mechanism is NHEJ rather than HRR, since the latter requires a sister

[1]Institute of Cancer and Genomic Sciences, University of Birmingham, Edgbaston, Birmingham B15 2TT, UK. [2]Neuroscience and Ophthalmology, Institute of Inflammation and Ageing, University of Birmingham, Edgbaston, Birmingham B15 2TT, UK. [3]Applied Medical Science College, Shaqra University, Addawadmi, Riyadh, Saudi Arabia. [4]Centre for Trauma Sciences Research, University of Birmingham, Edgbaston, Birmingham B15 2TT, UK.
*Corresponding author. Email: r.i.tuxworth@bham.ac.uk (R.I.T.); z.ahmed.1@bham.ac.uk (Z.A.)
†These authors contributed equally to this work.

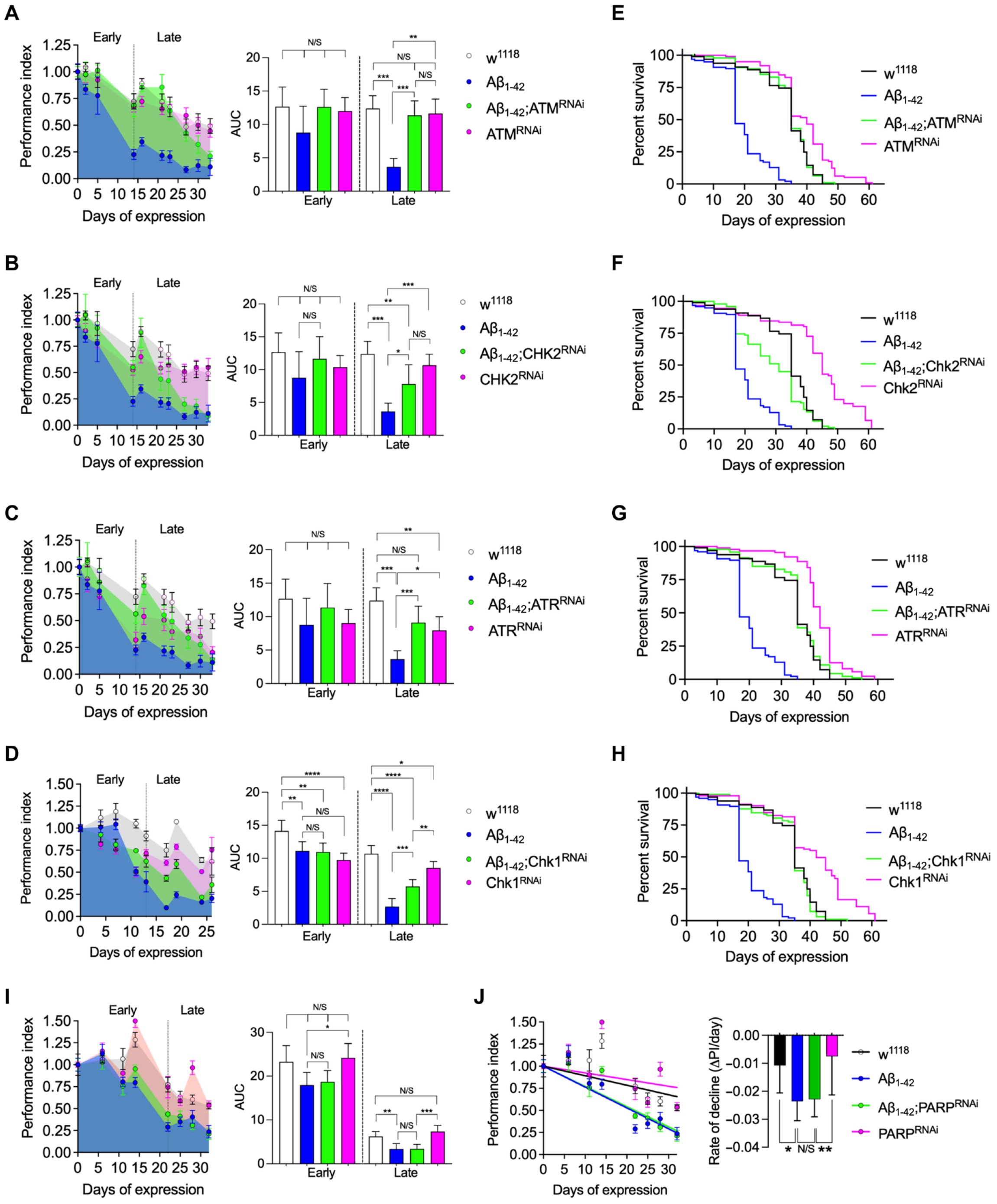

Fig. 1. Inhibition of Chk2 maintains neural function and increases survival in an amyloid toxicity model in *Drosophila*. (**A** to **E**) Longitudinal startle responses of *Drosophila* expressing amyloid beta ($A\beta_{1-42}$) in adult neurons. Knockdown by RNAi of (A) ATM (*tefu*), (B) Chk2 (*lok*), (C) ATR (*mei-41*), and (D) Chk1 (*grp*) all significantly slow the rate of decline of the startle response and survival induced by $A\beta_{1-42}$. For comparisons, the startle responses for each genotype were converted to a performance index and normalized to the initial response. The longitudinal responses were split into early and late phases, and the AUCs were compared by ANOVA [left panels for (A) to (D) and (I)]. ****$P < 0.0001$, ***$P = 0.001$, **$P = 0.01$, and *$P = 0.05$, ANOVA with Dunnett's post hoc test. $n = 5$ for all genotypes. Survival is also significantly enhanced after knockdown of (E) ATM, (**F**) Chk2, (**G**) ATR, and (**H**) Chk1 ($P < 0.0001$ in each case; log-rank analysis). (**I**) Knockdown of PARP important in DNA single-strand break repair pathways has no effect on the startle response using either the (I) AUC method or by comparing (**J**) rates of decline in performance index by simple linear regression. N/S, nonsignificant.

chromatid as a template. ATR signaling in DSB repair is primarily targeted at the HRR pathway. It is further central to the resolution of replication stress, which should not be present unless neurons have aberrantly reentered the cell cycle. However, knockdown of both ATR (Fig. 1C) and Chk1 (Fig. 1D) did show a significant protective effect (Fig. 1C), potentially indicating some cross-talk between the ATM and ATR pathways in *Drosophila* or that some neurons become polyploid in the adult CNS (*14*). Knockdown of ATM-Chk2 and ATR-Chk1 also significantly extended the lifespan of $A\beta_{1\text{-}42}$-expressing flies (Fig. 1, E to H). The protective effect appears to be specific to DSB signaling since knockdown of poly(ADP-ribose) polymerase 1 (PARP1), which is central to mechanisms of single-strand break repair, had no effect on the toxicity of $A\beta_{1\text{-}42}$ (Fig. 1, I and J).

Inhibition of Chk2 and not Chk1 promotes dorsal root ganglion neuron survival and neurite outgrowth in vitro

The ATM-Chk2 and ATR-Chk1 pathways activated by DSBs are conserved between *Drosophila* and mammals (*15*, *16*). Since DSBs are also a feature of the pathophysiology of acute neurotrauma, we asked whether inhibiting Chk1 and Chk2 activity would be neuroprotective in models of SCI and optic nerve injury (*17*, *18*). In an in vitro model of SCI, where adult primary rat dorsal root ganglion neurons (DRGN) are cultured in the presence of inhibitory concentrations of CNS myelin extracts and 5-fluro-2-deoxyuridine (5-FDU) to limit glial proliferation, Chk2 was phosphorylated at the ATM target residue, Thr^{68} ($pChk2^{T68}$), and at an autophosphorylation site, Thr^{383} ($pChk2^{T383}$), required for activation (Fig. 2, A and B). Treatment with the specific Chk2 inhibitor, CCT241533 (termed Chk2i here), suppressed Chk2 phosphorylation at both Thr^{68} and Thr^{383} (Fig. 2, A and B), and improved DRGN survival from 40% in Neurobasal-A (NBA)–treated controls to 90% in Chk2i-treated wells (Fig. 2C). Chk2i also stimulated neurite outgrowth in DRGN over and above that observed for the positive control, fibroblast growth factor 2 (FGF2), in terms of both the number of DRGN with neurites (42% versus 82%) (Fig. 2D) and the mean neurite length (180 μm versus 520 μm) (Fig. 2, E and F). In contrast, treatment with the Chk1 inhibitor, LY2603618 (termed Chk1i here), had no effect on DRGN survival or neurite outgrowth (Fig. 2, C to F).

The observations with Chk1i and Chk2i were confirmed in this in vitro model of SCI by specific inhibition of Chk1 and Chk2 using both small interfering RNA (siRNA) and short hairpin–mediated RNA (shRNA) to Chk1 (siChk1/shChk1) and Chk2 (siChk2/shChk2). For example, siRNA-mediated knockdown of Chk2 mRNA (Fig. 3A) and protein (Fig. 3, B and C) promoted significant DRGN survival (Fig. 3D), increased the proportion of DRGN with neurites (Fig. 3E), and increased the mean DRGN neurite length (Fig. 3, F and G). Likewise, shRNA-mediated knockdown of Chk2 (fig. S1A) and not Chk1 (fig. S1B) also promoted significant DRGN survival (fig. S1C), increased the proportion of DRGN with neurites (fig. S1D), and increased the mean DRGN neurite length (fig. S1, E and F).

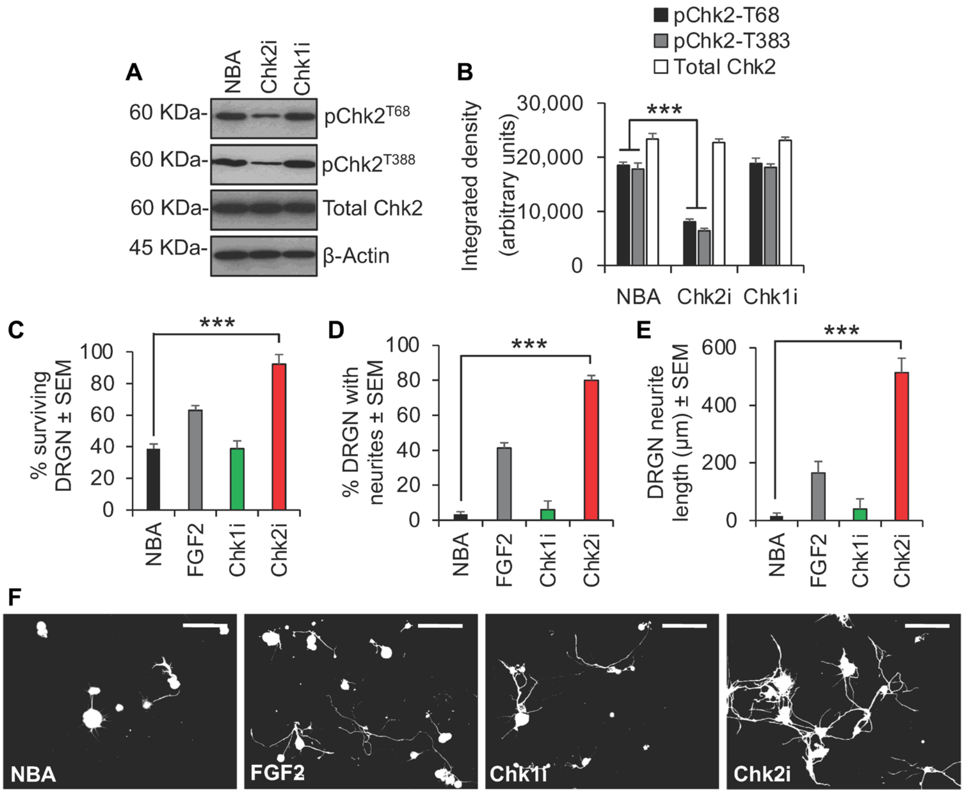

Fig. 2. Inhibition of Chk2 promotes DRGN survival and neurite outgrowth in vitro. (**A**) Western blot and (**B**) densitometry to show that Chk2i suppresses pChk2T68 and pChk2T383 in DRGN cultures. ***$P = 0.00011$, ANOVA with Dunnett's post hoc test. (**C**) Representative images after treatment with Chk2i and quantification to show that Chk2i enhances (**D**) % surviving DRGN, (**E**) % DRGN with neurites, and (**F**) the mean neurite length. FGF2 was used as a positive control. ***$P = 0.0001$, ANOVA with Dunnett's post hoc test. $n = 3$ wells per treatment, three independent repeats (total, $n = 9$ wells per treatment). Scale bars in (F) = 50 μm.

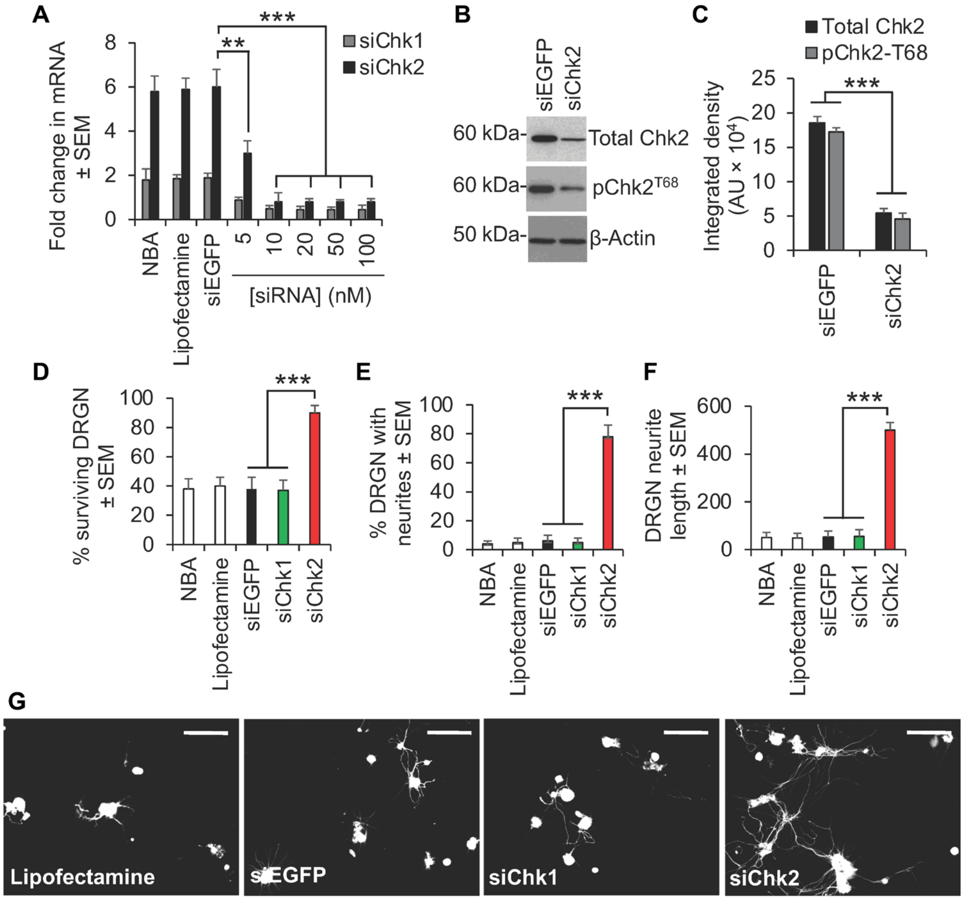

Fig. 3. Inhibition of Chk2 using siRNA confirms Chk2i results. Knockdown of Chk2 (**A**) mRNA and (**B** and **C**) protein with siRNA to Chk2 (siChk2) and not siRNA to Chk1 (siChk1) confirms significant (**D**) DRGN survival and neurite outgrowth in terms of (**E**) the number of DRGN with neurites and (**F**) the mean neurite length. (**G**) Representative images to show neurite outgrowth in DRGN after treatment with Lipofectamine (siRNA transfection reagent), siEGFP (nonspecific siRNA), siChk1, and siChk2. FGF2 was used as a positive control. ****P* = 0.0001 and ***P* = 0.001, ANOVA with Dunnett's post hoc test. *n* = 3 wells per treatment, three independent repeats (total, *n* = 9 wells per treatment). Scale bars in (G) = 50 μm.

These results demonstrate that suppression of Chk2 and not Chk1 promotes significant DRGN survival and neurite outgrowth and are consistent with our results from *Drosophila* and highlight Chk2 as the key target molecule.

Inhibition of Chk2 and not Chk1 promotes axon regeneration/plasticity and functional recovery after SCI in vivo

We extended our findings to ask whether suppression of Chk2 activity promotes axon regeneration/plasticity and functional recovery in vivo using the translationally relevant in vivo model of T8 dorsal column (DC) crush injury model of SCI in rats (*19*, *20*). Chk2 was phosphorylated at both Thr^{68} and Thr^{383} at 28 days after injury, but this was abolished by weekly intrathecal injections of Chk2i (Fig. 4, A and B). No changes in Chk1 phosphorylation were induced by DC injury or by Chk2i treatment (Fig. 4, A and B). Chk2i promoted significant DC axon regeneration/sprouting by 6 weeks after injury at all distances rostral to the lesion site despite the presence of spinal cord cavities, with 23.7% of growth-associated protein 43 $(GAP43)^{+}$ axons regenerating 6 mm rostral to the lesion site (Fig. 4, C and D). In contrast, Chk1i and vehicle-treated rats showed no axon regeneration beyond the lesion site (Fig. 4, C and D). We used GAP43 to demonstrate DC axon regeneration/sprouting, since in our hands, Cholera toxin B labeling does not work in the rat (*18*), while it is well established that GAP43 is highly expressed during periods of active growth and synaptogenesis (*21*–*23*).

The improvements in axon regeneration after inhibition of Chk2 also correlated improved electrophysiological recovery across the lesion site at 6 weeks after SCI. For example, DC injury abolished the normal compound action potential (CAP) trace observed in sham-treated controls, but Chk2i returned a significant CAP trace (Fig. 4E), CAP amplitude (Fig. 4F), and CAP area (Fig. 4G) in treated animals, while Chk1i had no effect. Likewise, the tape sensing and removal and ladder crossing tests showed that Chk2i resulted in significant improvements in sensory (Fig. 4H) and locomotor function (Fig. 4I) such that animals were indistinguishable from sham-treated controls by 3 weeks after SCI. Once again, Chk1i had no effect on sensory or locomotor function, and as with DC + vehicle–treated rats, a significant deficit remained for the full 6-week duration. These results demonstrate that Chk2i after SCI promotes significant DC axon regeneration along with improvements in electrophysiological, sensory, and locomotor function.

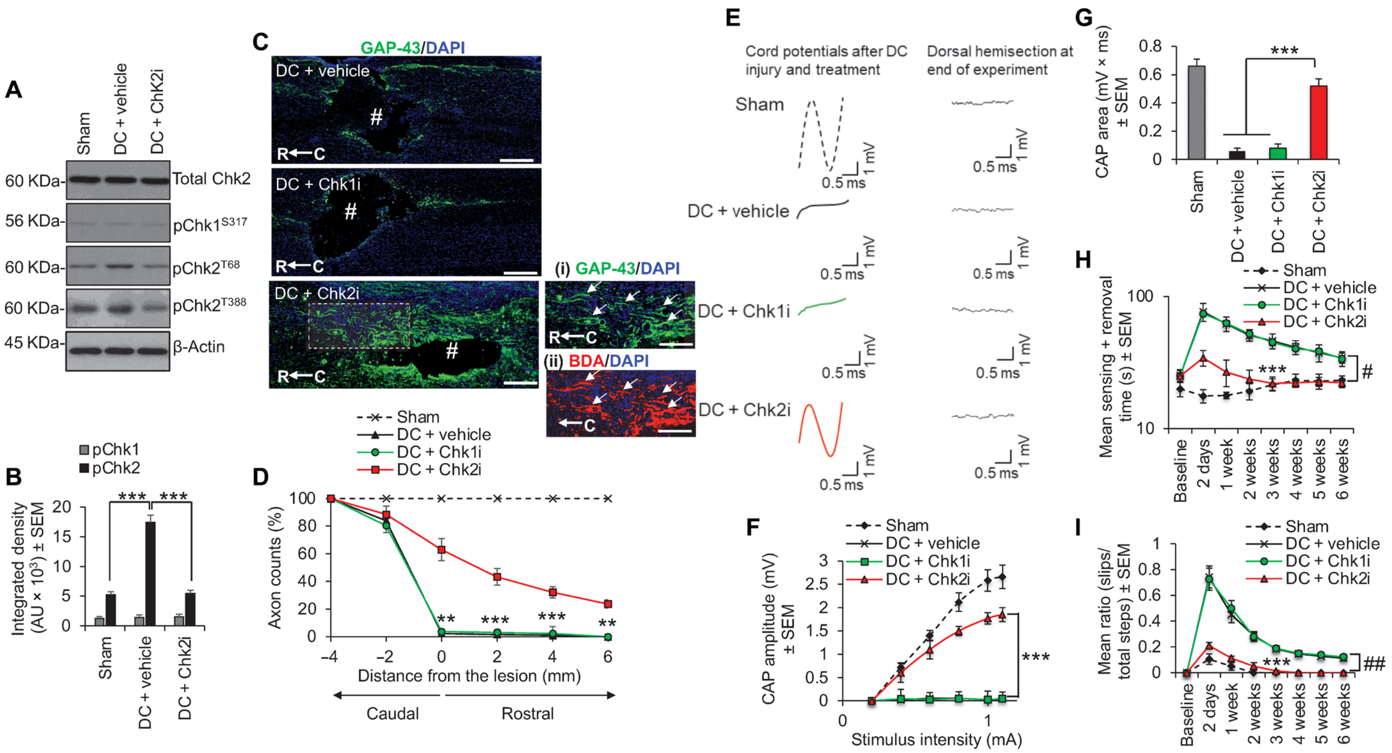

Fig. 4. Inhibition of Chk2 promotes DC axon regeneration in vivo. (**A**) Western blot and (**B**) densitometry to show that Chk2i significantly suppresses pChk2T68 and pChk2T383 levels after DC injury without affecting pChk1 levels. (**C**) Many GAP43^{+} axons [green (DAPI^{+} nuclei = blue)] were observed in DC + Chk2i regenerating through the lesion site and into the rostral cord (boxed region = high power view of GAP43^{+} axons in the rostral cord) despite the presence of a large cavity (#), while few GAP43^{+} axons were present beyond the lesion site in DC + vehicle– and DC + Chk1i–treated spinal cords. GAP43 staining [C (i)] was confirmed with biotynilated dextran amine (BDA) tracing of the same axons [C (ii)]. (**D**) Quantification of the number of GAP43^{+} axons at distances caudal and rostral to the lesion site showing significant proportions of axons regenerating up to 6 mm beyond the lesion epicenter. Scale bars in (C), 200 μm. $^{**}P = 0.0012$ and $^{***}P = 0.0001$, ANOVA with Dunnett's post hoc test. $n = 6$ nerves per treatment, three independent repeats (total, $n = 18$ nerves per treatment). (**E**) Spike 2 software–processed CAP traces from representative sham controls, DC + vehicle–, DC + Chk1i–, and DC + Chk2i–treated rats at 6 weeks after DC injury and treatment. Dorsal hemisection at the end of recording ablated all CAP traces. (**F**) Negative CAP amplitudes and (**G**) CAP area at different stimulation intensities were both significantly attenuated in DC + vehicle– and DC + Chk1i–treated rats but were restored in DC + Chk2i–treated rats [$P = 0.0001$, one-way ANOVA with Dunnett's post hoc test (main effect)]. (**H**) Mean tape sensing/removal times and (**I**) mean error ratio to show the number of slips versus total steps are both restored to normal 3 weeks after treatment with Chk2i [$^{***}P = 0.0001$, independent sample *t* test (DC + vehicle versus DC + Chk2i at 3 weeks)], while a significant deficit remains in DC + vehicle– and DC + Chk1i–treated rats (#$P = 0.00014$, generalized linear mixed models; ##$P = 0.00011$, linear mixed models over the whole 6 weeks). $n = 6$ rats per treatment, three independent repeats (total, $n = 18$ rats per treatment).

Prexasertib, a potent Chk1/Chk2 inhibitor, promotes DRGN neuroprotection in vitro and improves functional recovery after SCI in vivo

To start translating our findings and identify potential treatment options for patients, we tested prexasertib (LY2606368). Prexasertib is a potent inhibitor of both Chk1 and Chk2 [median inhibitory concentration (IC_{50}) of 8 nM for Chk2] but, crucially, has already been used in phase 2 clinical trials in cancer (*24*). In an in vitro serum withdrawal model of DRGN, ~60% DRGN died within 5 days in culture in the absence of serum, while the presence of serum promoted nearly 80% DRGN survival, as expected (*25*) (fig. S4A). However, inhibition of Chk2 using Chk2i or prexasertib in the absence of serum also supported >80% DRGN survival, while Chk1i had no neuroprotective effects (fig. S4A). Both the number of DRGN with neurites and the mean neurite length were increased significantly by either Chk2i and prexasertib treatment versus DMEM + serum or DMEM alone (fig. S4, B to D). These results demonstrate that Chk2 inhibition rescues DRGN survival and neurite outgrowth in a serum withdrawal model in vitro.

We then tested prexasertib in our in vivo DC injury model of SCI. Prexasertib treatment significantly suppressed pChk2^{T68} levels (Fig. 5, A and B) and restored a significant CAP trace (Fig. 5C), CAP amplitude (Fig. 5D), and >80% of the CAP area observed in sham-treated controls (Fig. 5E). These electrophysiological improvements correlated with significant improvements in sensory (Fig. 5F) and locomotor performance (Fig. 5G). The effects of prexasertib were due to its inhibitory activity on Chk2 as there was no response to treatment with a specific Chk1i.

Patients with SCI are usually stabilized for 12 to 24 hours after injury before treatments are administered. To ask whether prexasertib would work in a clinically relevant time frame, we waited 24 hours after SCI before administering treatment. Electrophysiology demonstrated similar recovery of CAP traces (Fig. 6A), CAP amplitude (Fig. 6B), and CAP area (Fig. 6C), as observed with prexasertib treatment immediately after injury (see Fig. 4). There was also a similar improvement in sensory (Fig. 6D) and locomotor function (Fig. 6E) with prexasertib treatment, as seen in those treated immediately after injury (see Fig. 4). These results demonstrate that

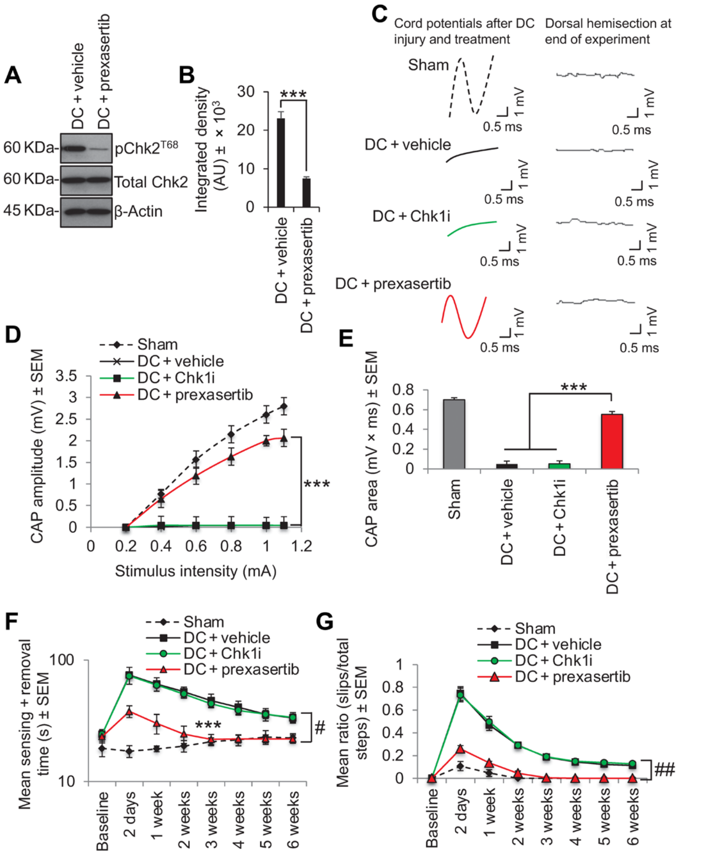

Fig. 5. Inhibition of Chk2 using prexasertib promotes functional recovery after DC injury in vivo. (**A**) Western blot and (**B**) densitometry to show that prexasertib significantly suppresses pChk2^{T68} levels after DC injury without affecting total Chk2 levels. (**C**) Spike 2 software–processed CAP traces from representative sham controls, DC + vehicle–, DC + Chk1i–, and DC + prexasertib–treated rats at 6 weeks after DC injury and treatment. Dorsal hemisection at the end of recording ablated all CAP traces. (**D**) Negative CAP amplitudes and (**E**) CAP area at different stimulation intensities were both significantly attenuated in DC + vehicle– and DC + Chk1i–treated rats but were restored in DC + prexasertib–treated rats [$P = 0.0001$, one-way ANOVA with Dunnett's post hoc test (main effect)]. (**F**) Mean tape sensing/removal times and (**G**) mean error ratio to show the number of slips versus total steps are both restored to normal 3 weeks after treatment with Chk2i [$^{***}P = 0.0001$, independent sample *t* test (DC + vehicle versus DC + Chk2i at 3 weeks)], while a significant deficit remains in DC + vehicle– and DC + Chk1i–treated rats ($\#P = 0.00012$, generalized linear mixed models; $\#\#P = 0.00014$, linear mixed models over the whole 6 weeks). $n = 6$ rats per treatment, three independent repeats (total, $n = 18$ rats per treatment).

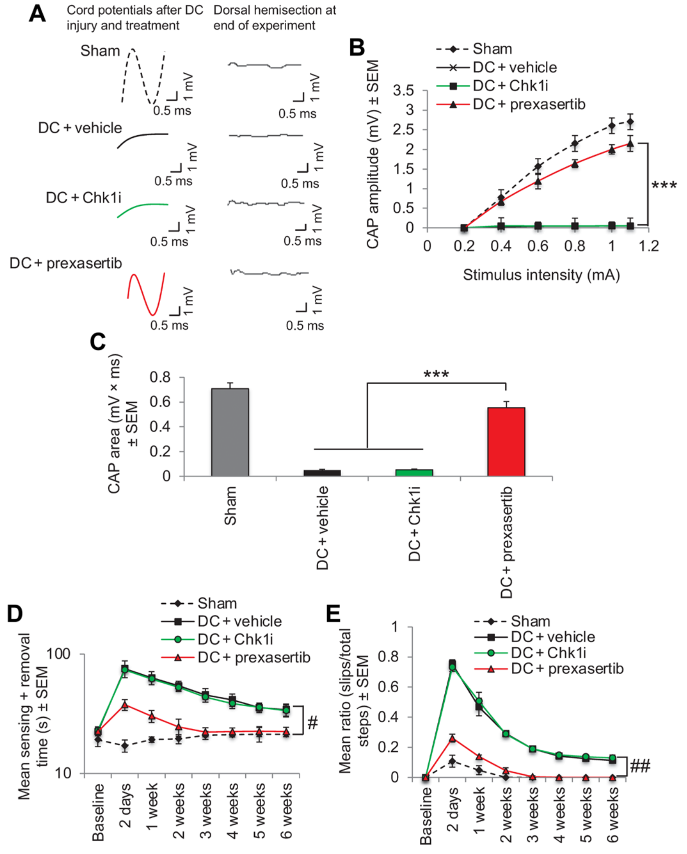

Fig. 6. Twenty-four–hour delayed treatment with prexasertib is equally as effective as immediate treatment in restoring electrophysiological, sensory, and locomotor recovery after DC injury. (**A**) Spike 2 software–processed CAP traces at 6 weeks after DC injury from representative sham controls, DC + vehicle–, DC + Chk1i–, and DC + prexasertib–treated rats. (**B**) Negative CAP amplitudes and (**C**) CAP areas were significantly attenuated in DC + vehicle– and DC + Chk1i–treated rats but were restored in DC + prexasertib–treated rats [$P < 0.0001$, one-way ANOVA (main effect)]. (**D**) Mean tape sensing and removal times were restored to normal 3 weeks after treatment with shChk2, while a significant deficit remained in DC + vehicle– and DC + Chk1i–treated rats ($\#P < 0.00014$, generalized linear mixed models over the whole 6 weeks). (**E**) Mean error ratio to show the number of slips versus total number of steps in the horizontal ladder walking test also returns to normal 3 weeks after treatment with prexasertib with a deficit remaining in DC + vehicle– and DC + Chk1i–treated rats ($\#\#P < 0.00011$, linear-mixed models over the whole 6 weeks). $n = 6$ rats per treatment per test, three independent repeats (total, $n = 18$ rats per treatment per test).

treatment of SCI with prexasertib either immediately or within 24 hours after injury elicits the same functional benefits.

Dose-dependent effects of prexasertib in improving function after SCI

Although we obtained nearly 70% inhibition of pChk2 with a dose of 2 μg of prexasertib, we performed a dose de-escalation study in our SCI model to determine whether lower doses could elicit similar functional benefits. We observed that treatment with 0.2 and 0.02 μg of prexasertib suppressed pChk2 levels significantly by 54 and 31%, respectively, when compared with DC + vehicle–treated animals (Fig. 7, A and B). Both lower doses were able to restore a significant CAP trace (Fig. 7C) and CAP amplitude (Fig. 7D) as well as restore the CAP area to within 75 and 61%, respectively (Fig. 7E), compared with sham-treated animals (to compare: 2 μg of prexasertib restored 79% of the CAP area compared with sham-treated animals).

There was little or no differences in sensory and locomotor recovery after treatment of SCI animals with 0.2 μg of prexasertib compared with 2 μg (Fig. 7, F and G), and even with the lowest dose of 0.02 μg, recovery of sensory and locomotor function was only marginally slower, taking 1 week longer for the recovery to be indistinguishable from sham-treated control animals (Fig. 7, F and G). These results demonstrate that only a ~30% inhibition of pChk2 by prexasertib is sufficient to elicit significant functional benefits in SCI-treated animals.

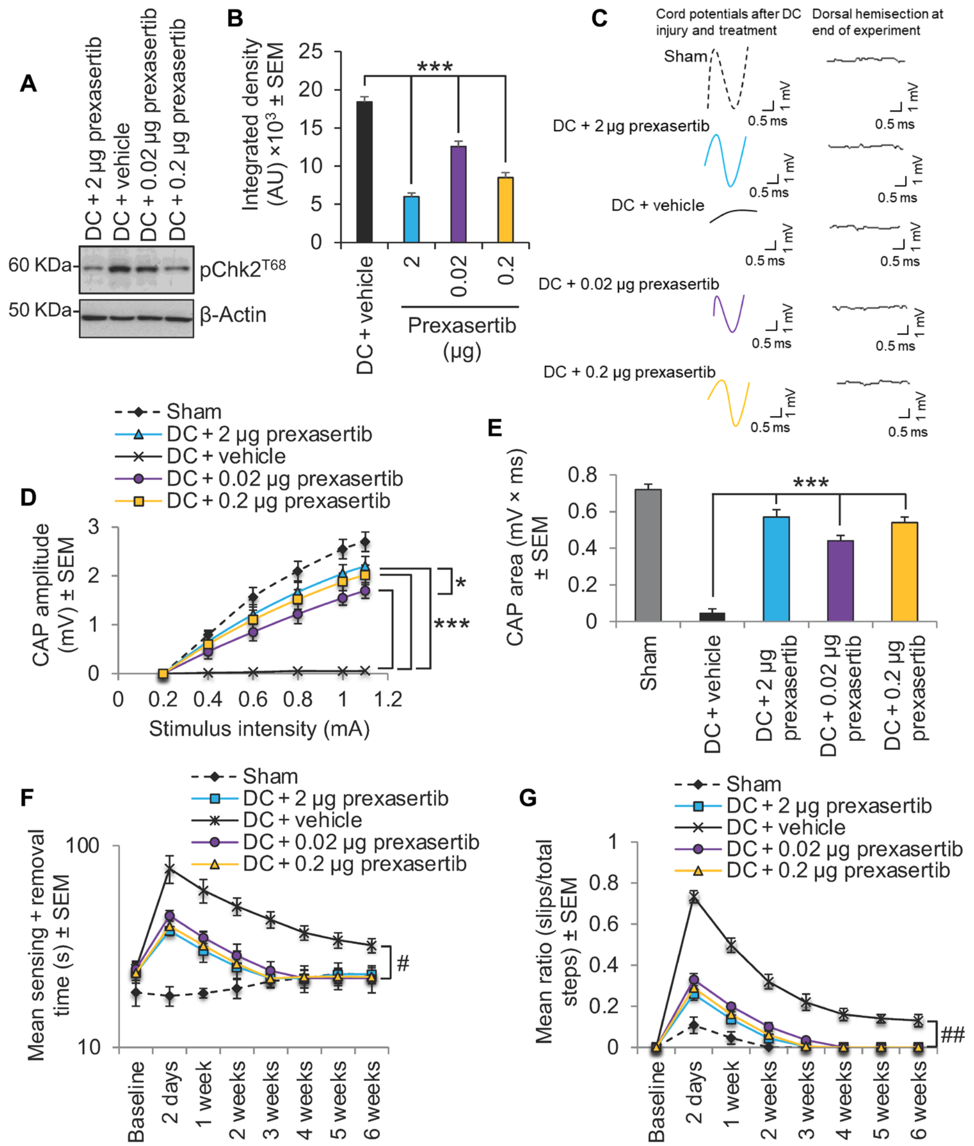

Fig. 7. Dose de-escalation study with prexasertib. (**A**) Western blot and (**B**) densitometry to show that lower doses (0.2 and 0.02 μg) of prexasertib than that required to cause maximal suppression of pChk2 levels (i.e., 2 μg) were still able to significantly suppresses pChk2T68 levels after DC injury. (**C**) Spike 2 software–processed CAP traces from representative sham controls, DC + 2 μg prexasertib–, DC + vehicle–, DC + 0.02 μg prexasertib–, and DC + 0.2 μg–treated rats at 6 weeks after DC injury and treatment. Dorsal hemisection at the end of recording ablated all CAP traces. (**D**) Negative CAP amplitudes and (**E**) CAP area at different stimulation intensities were both significantly attenuated in DC + vehicle–treated rats but were dose-dependently restored in DC + prexasertib–treated rats [$P = 0.00011$, one-way ANOVA with Dunnett's post hoc test (main effect)]. (**F**) Mean tape sensing/removal times and (**G**) mean error ratio to show the number of slips versus total steps are both restored to normal 3 weeks after treatment with 2 and 0.2 μg of prexasertib but were restored to normal 4 weeks after DC injury and treatment with 0.02 μg of prexasertib (#$P = 0.00012$, generalized linear-mixed models and ##$P = 0.00014$, linear-mixed models over the whole 6 weeks). A significant deficit remains in DC + vehicle–treated rats. $n = 6$ rats per treatment, three independent repeats (total, $n = 18$ rats per treatment).

Prexasertib is neuroprotective and axon regenerative after optic nerve injury

Acute CNS trauma to the optic nerve by a crush injury normally promotes significant retinal ganglion cell (RGC) apoptosis within 2 weeks after injury (*26*, *27*). Therefore, we asked whether Chk2 inhibition using prexasertib can not only be neuroprotective but also axon regenerative in this second model of CNS trauma. We observed that prexasertib (to inhibit Chk2) and not a specific inhibitor of Chk1 promoted significant RGC survival (Fig. 8A) and neurite outgrowth in vitro, increasing the number of RGCs with neurites (Fig. 8B) and the mean neurite length (Fig. 8, C and D). Weekly intraocular injections of prexasertib to ONC-injured rats in vivo also promoted >90% RGC survival (Fig. 8, E and F) and significant RGC axon regeneration (Fig. 8, G and H), which was accompanied by significant improvement (>83%) in RGC function, as measured by flash electroretinography (ERG) (Fig. 8, I and J). These results demonstrated that prexasertib is also neuroprotective and axon regenerative in this second model of CNS trauma.

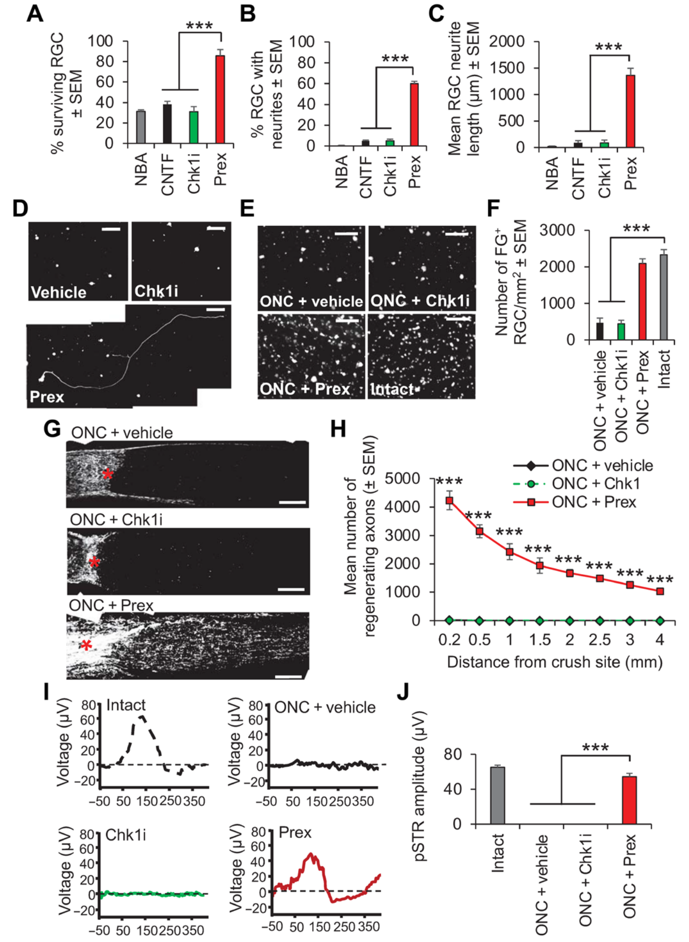

Fig. 8. Chk2 inhibition using prexasertib prevents RGC apoptosis and stimulates neurite outgrowth/axon regeneration after 4 days in vitro and 24 days after ONC in vivo. (**A**) Preoptimized concentration of prexasertib (Prex) in culture at 4 days significantly enhanced RGC survival compared with control NBA, positive control CNTF (preoptimized), or Chk1i. Prexasertib also enhanced neurite outgrowth, increasing the (**B**) % RGC with neurites and the (**C**) mean neurite length compared to all other treatment groups. (**D**) Representative images from RGCs treated with vehicle, Chk1i, and prexasertib. Scale bars, 100 μm. $n = 3$ wells per treatment per test, three independent repeats (total, $n = 9$ wells per treatment per test). (**E**) Representative images of FG-labeled RGCs in retinal wholemounts at 24 days after ONC in vivo and (**F**) quantification show that prexasertib significantly protected RGCs from death. Scale bars in (E), 50 μm. (**G**) Representative images of longitudinal sections of optic nerves at 24 days after ONC stained for GAP43 from ONC + vehicle, ONC + Chk1i, and ONC + prexasertib and (**H**) quantification to show that prexasertib significantly enhanced RGC axon regeneration through the lesion site (*) and into the distal optic nerve segment [$n = 6$ nerves per condition, three independent repeats (total, $n = 18$ nerves per condition)]. $P = 0.0001$, ANOVA with Dunnett's post hoc test. Scale bars in (G), 200 μm. (**I**) The normal ERG trace in intact rats were ablated in ONC + vehicle and ONC + Chk1i treatment, but a significant ERG trace is restored in ONC + prexasertib–treated rats. (**J**) Mean pSTR amplitudes demonstrate that prexasertib restored significant visual function after ONC. $P = 0.0001$, ANOVA with Dunnett's post hoc test. Note: In (H), ONC + vehicle and ONC + Chk1 values are similar and hence appear superimposed.

DISCUSSION

Here, we show that Chk2 is a novel therapeutic target to prevent functional decline in acute and chronic neurological disease. We show that targeting the Chk2 pathway suppresses neurodegeneration in *Drosophila* models of amyloid toxicity, and administering clinically relevant Chk2 inhibitors to rats, following SCI and optic nerve injury, results in significant axon regeneration/sprouting and a marked recovery of lost sensory, motor, and visual function.

DSBs in DNA accumulate in most, potentially all, neurological diseases, including chronic disorders such as Alzheimer's disease, amyotrophic lateral sclerosis, and after acute stroke or physical trauma to the CNS (*7*, *9*–*11*). The cause of DSBs might possibly be ROS based in each disorder, but regardless of the cause, neurons appear unable to fully repair the DSBs in these pathological scenarios, with resulting chronic activation of the DDR.

We assumed that postmitotic neurons in G_0 of the cell cycle would attempt to repair the DSBs via NHEJ as a sister chromatid required for HRR should not be present. In this case, we were expecting that the ATM-Chk2 pathway would be a better target for knockdown than the ATR-Chk1 pathway, given that ATM regulates both HRR and NHEJ and the G_1/M checkpoint, while ATR-Chk1 is primarily involved in the resolution of replication stress, HRR signaling, and the G_2/M checkpoint. It was unexpected therefore that genetic targeting of either the ATM or ATR pathway suppressed the toxicity of $A\beta_{1-42}$ oligomers in *Drosophila* and extended their lifespan. This may reflect some crossover between the ATM-Chk2 and ATR-Chk1 pathways in flies or the possible availability of HR templates: either because some neurons in adult flies become polyploid (*14*) or potentially due to inappropriate cell cycle reentry by neurons. Cell cycle reentry can result from chronic activation of the DDR and trigger apoptosis or senescence, as has been reported in postmortem brains from patients with Alzheimer's disease (*28*, *29*).

The picture was much clearer in our two models of acute CNS injury in rats. The use of tool and clinically relevant inhibitors of Chk1 and Chk2 in both SCI and optic nerve injury models showed that inhibiting only the Chk2 pathway promoted functional recovery after injury, but inhibiting the Chk1 pathway had no effect. Following SCI, Chk2 inhibition promoted axon regeneration/sprouting and electrophysiological, sensory, and locomotor recovery after injury. In the optic nerve injury model, Chk2 inhibition also promoted axon regeneration and electrophysiological functional recovery, but, in this model, Chk2 inhibition also protects RGCs from apoptosis in vivo. Optic nerve crush (ONC) injury results in rapid death of RGCs such that by 2 weeks, 80% of RGCs die by apoptosis (*26*, *27*, *30*). This is probably because RGC axons are unidirectional and retrogradely transport neurotrophic factors from the target organ, and once the optic nerve is crushed, RGCs lose their neurotrophic supply and die. The additional neuroprotective effect is not critical in our SCI model, where the widespread death of dorsal root ganglion (DRG) neurons does not occur (*25*). It is likely that DRGN survival in vivo is probably maintained through neurotrophic factor supply from alternative ascending and descending pathways. However, we were able to show significant neuroprotective effects of Chk2 inhibition on DRGN in an in vitro model of serum withdrawal–induced DRGN apoptosis. Therefore, the benefits of Chk2 inhibition on DRGN survival are likely to be helpful in the in vivo scenario.

Chk2 is phosphorylated by the ATM, initiating subsequent Chk2 autophosphorylation and activation (*31*, *32*). ATM acts as a master regulator of the DDR, and we and others have demonstrated neuroprotective effects of targeting ATM. Caffeine, a nonspecific inhibitor of ATM and other phosphatidylinositol 3-kinase family members, protects against etoposide-induced DNA damage and cell death in neurons in vitro, and genetic reduction of ATM gene dosage is neuroprotective in mouse models of Huntington's disease (*33*, *34*).

However, ATM has >700 potential targets and is activated by multiple other factors in addition to DBSs, including ROS, and it regulates several downstream cellular processes in addition to the DDR. This broad range of activity potentially makes it a difficult therapeutic target. Chk2, on the other hand, has far fewer targets (~25) and regulates fewer downstream processes (*31*, *32*), and it may prove to be a better therapeutic target for CNS injury, especially SCI.

The clinically relevant Chk2 inhibitors used in this study, prexasertib and CCT245133, are both small-molecule inhibitors with a low IC_{50} for Chk2 and developed for use in the treatment of cancer. CCT241533 was developed by Cancer Research UK, with an oral bioavailability of 38 to 54%, and preliminary pharmacokinetic/pharmacodynamic studies demonstrate that it is well tolerated in mouse studies and shows efficacy in a tumor xenograft model (www.cancerresearchhorizons.com/licensing-opportunities/chk2-inhibitor-programme). Prexasertib (LY2606368) was developed by Eli Lilly as a Chk1/2 inhibitor with an IC_{50} of 8 nM for Chk2 and has been used in phase 2 clinical trials in prostate cancer (NCT02407054) and in 18 other studies that include solid tumors and blood cancers (e.g., NCT03057145, NCT02555644, and NCT03735446). Hence, prexasertib is a good candidate for repurposing to treat SCI. There are currently no fully restorative therapies for SCI, and it is a major area of unmet clinical need. The only drug that is currently approved by the Food and Drug Administration in SCI is pregabalin (Lyrica) for the treatment of SCI-induced neuropathic pain, but this has no prorestorative effects. Several experimental therapies have been developed, including anti-Nogo antibodies, the Rho antagonist, Cethrin, and mesenchymal stem cells transplants, but all of these are in early development (*35*–*37*). In contrast, prexasertib has the potential to be progressed rapidly to clinical trials (*38*).

Our DC injury model of SCI faithfully recapitulates many aspects of the human SCI (compression or contusion) pathology including cavity, cyst, and syrinx formation and cord disruption (*39*–*41*). These account for >80% of all SCI in humans. Cavitation is not present in mouse models of SCI. Crucially for translation, our experiments demonstrated that Chk2 inhibitors were equally effective at improving sensory and motor function when delivered immediately after injury or up to 24 hours after SCI. This is directly relevant to the treatment of human patients since most new cases attend emergency care immediately but may need stabilizing for up to 24 hours before drugs can be administered. In addition, it appears that only 30% inhibition of pChk2 is required for significant functional recovery, suggesting that low doses of prexasertib will suffice. Drugs can be delivered directly to the injury site via intrathecal injections, as we used in our model, or, as is the case with prexasertib, can also be given by subcutaneous (*42*, *43*) or intravenous injections (*44*). Intrathecal delivery has obvious advantages as it gives direct access to the CNS, often requiring lower doses of drugs and with greater efficiency than other routes of administration, limiting unwanted side effects (*45*). It remains to be seen whether inhibiting the DDR in dividing cells in the CNS, such as glia, is detrimental or not but will require caution when used in humans.

Prexasertib was also effective in a second model of CNS injury: the ONC injury model (*17*, *46*). The model is directly relevant to glaucoma and other eye trauma or eye diseases where the death of RGC occurs and is an excellent model to test neuroprotective therapies. ONC induces the rapid death of RGCs, an effect orchestrated by activation of caspase-2 (*26*). While caspase-2 inhibition prevents apoptosis, it does not however permit regeneration of RGC axons as they appear to be regulated by different pathways (*26*, *27*, *30*). In contrast, Chk2 inhibition promoted greater than 90% RGC neuroprotection while also stimulating significant RGC axon regeneration, which appeared to be as effective as our previous results using Mre11 or ATM inhibitors (*12*).

In our hands, prexasertib promoted RGC axon regeneration to a greater extent than other treatments in the optic nerve injury model (*27*, *47*–*49*). For example, lens injury and oncomodulin promoted the regeneration of fewer than 1000 RGC axons beyond 1 mm at 21 days after ONC (*47*, *48*). However, Chk2 inhibition using prexasertib promoted the regeneration of greater than 2400 RGC axons beyond 1 mm with >1000 axons detected at 4 mm beyond the lesion center. These levels of RGC axon regeneration after prexasertib treatment led to restitution of >80% of the pSTR amplitudes and, hence, visual function. Our results demonstrated the utility of prexasertib as an effective therapy to promote both neuroprotection and RGC axon regeneration in the eye.

In conclusion, this study shows that Chk2 inhibitors, including the repurposing candidate prexasertib, promote neuroprotection, axon regeneration, and marked restoration of lost function after CNS injury. Inhibition of Chk2 is an exciting and clinically feasible new approach with potential to address the unmet clinical needs of patients with acute neurotrauma.

MATERIALS AND METHODS

Experimental design

The goal of this study was to elucidate the role of ATM-Chk2 in CNS trauma. We used *Drosophila* to genetically knock down ATM-Chk2 or ATR-Chk1 to assess the impact on performance in a movement assay and by observing lifespan. We then not only used the rat DC crush injury model of SCI (*19*) as our primary model but also used the ONC injury model as a second model of CNS injury (*17*). Cell culture experiments using primary adult rat DRGN and retinal cells containing RGCs and inhibitors/siRNA/shRNA were used in mechanistic studies. In vivo sample sizes were determined at the outset and on the basis of power calculations derived from previous similar experiments in our laboratories. Animals were randomly assigned to treatment groups roughly containing equal numbers of male and female animals, and experimenters were masked to the treatment and procedural conditions. Animal tissue samples were all processed together and analyzed to prevent batch effects. All animals were housed in groups of four animals per cage in the same facility, and the number of biological replicates is indicated in the figure legends. No animals were excluded in this study nor were sample or data points omitted, for any reason.

Rats

All experiments in rats were licensed by the UK Home Office and ethically approved by the University of Birmingham's Animal Welfare and Ethical Review Board. All animal surgeries were carried out in strict accordance to the guidelines of the UK Animals Scientific Procedures Act, 1986 and the Revised European Directive 1010/63/EU and conformed to the guidelines and recommendation of the use of animals by the Federation of the European Laboratory Animal Science Associations (FELASA). Experiments in the eye and optic nerves also conformed to the ARVO statement for use of animals in research and the Animal Research: Reporting of in vivo Experiments (ARRIVE) guidelines. Adult male and female 6- to 8-week-old

Sprague-Dawley rats weighing 170 to 220 g (Charles River, Margate, UK) were used in all experiments. Animals were housed in a standard facility, kept on a 12-hour light/12-hour dark cycle with daytime luminance of 80 lux, fed, and watered ad libitum. Pre- and post-operative analgesia was provided as standard and as recommended by the named veterinary surgeon.

Drosophila methods

The *Drosophila* experiments were essentially performed as described in (*13*). Briefly, tandem Aβ_{1-42} peptides (*50*) were expressed in adult neurons under the control of *elav-Gal4* with expression suppressed until 7 to 10 days after eclosion by inclusion of Gal80^{ts}. Flies were maintained at 18°C to repress expression and then shifted to 27°C to induce expression. Longitudinal tracking of the startle response of flies was performed as in (*13*).

Comparison of startle performance

For each experiment, the longitudinal decline in performance index was separated into early and late phases, with the demarcation defined as the first day on which the performance of the Aβ_{1-42}-expressing flies had declined to below 0.5. Area under the curve (AUC) was calculated for the early and late phases for each genotype using Prism 9 and compared by analysis of variance (ANOVA). For the PARP experiment, a second method of comparison was used to confirm the lack of significance. In this case, linear regression lines were calculated for each genotype and compared by ANOVA, as described in (*13*).

Drosophila strains

Virgin females of the driver line: *w^{1118}*, *elav-Gal4^{c155}*; *Gal80^{ts}* were used for all crosses. UAS-tAb1-42 12-linker was described in (*50*) and was a gift of D. Crowther. UAS–RNA interference (RNAi) lines were obtained from the Bloomington *Drosophila* Stock Center: *tefu* (ATM): *TRiP.GL00138* (BL44417), *lok* (Chk2): *TRiP.GL00020* (BL35152), *mei*-41 (ATR): *TRiP.GL00284* (BL41934), and *grp* (Chk1): *TRiP.JF2588* (BL27277).

Adult rat DRGN and retinal cultures

Primary adult rat DRGN and retinal cultures (containing enriched populations of RGCs) were prepared as described by us previously (*17*, *51*). DRGN or retinal cells were cultured in Neurobasal-A (NBA; catalog no. 10888022, Invitrogen, Paisley, UK) at a plating density of either 500 per well or 125 × 10^3 cells per well in chamber slides (Beckton Dickinson, Oxford, UK) precoated with poly-D-lysine (100 μg/ml; Sigma-Aldrich, Poole, UK), respectively. To limit glial cell proliferation, 5-FDU (catalog no. 343333, Sigma) was used at 30 μM in both DRGN and retinal cultures (*51*). Positive controls included preoptimized FGF2 [10 ng/ml (*52*); catalog no. 450-33, Peprotech, London, UK] and CNTF [10 ng/ml (*17*); catalog no. 45-50, Peprotech) for DRGN and RGC cultures, respectively. Cells were cultured for 4 days in a humidified chamber at 37°C and 5% CO_2 before being subjected to quantitative reverse transcription polymerase chain reaction (qRT-PCR) or immunocytochemistry, as described below. Treatments were added in triplicate and repeated on three independent occasions.

Serum withdrawal model of primary adult DRGN survival

Primary adult rat DRGN were prepared as described above, except that dissociated DRGN were pelleted, resuspended in Dulbecco's modified Eagle's medium (DMEM; catalog no. 11965092, Invitrogen) at a plating density of 300 cells per well, and cultured with or without 10% fetal bovine serum (catalog no. A4766, Invitrogen), as described by us previously (*25*). To limit glial cell proliferation, 30 μM 5-FDU was used. Cells were cultured for 5 days in a humidified chamber at 37°C and 5% CO_2 before being subjected to immunocytochemistry for βIII-tubulin (1:400 dilution; catalog no. T4026, Sigma-Aldrich), counting DRGN survival and measuring DRGN neurite outgrowth, as described below. Treatments were added in triplicate and repeated on three independent occasions.

Chk inhibitor studies

All inhibitors were dissolved in dimethyl sulfoxide (catalog no. D8418, Sigma-Aldrich, Poole, UK) at a stock concentration of 10 mM for in vitro use. In preliminary experiments, the optimal concentration of CCT241533 (referred to as Chk2i from here; 10 μM; catalog no. CAY19178, Cambridge Bioscience, Cambridge, UK), BML-277 (5 μM; catalog no. 220486, Sigma-Aldrich, Poole, UK), and prexasertib (10 μM; catalog no. HY-18174A, Cambridge Bioscience, Cambridge, UK) that promoted DRGN/RGC survival and neurite outgrowth was determined. The Chk1 inhibitor LY2603618 (referred to from here as Chk1i; catalog no. 6454, Tocris, Oxford, UK) had no effect on DRGN/RGC survival at 1 to 50 μM, and hence, we used 20 μM, which was shown to induce DNA damage in a variety of human lung cancer cell lines including A549 and H1299 (*53*).

Transfection of DRGN cultures with siRNA/shRNA

ON-TARGETplus rat Chk1 shRNA (siChk1; catalog no. J-094741-09-0002) and Chk2 siRNA (siChk2; catalog no. J-096968-09-0002) were purchased from Dharmacon (Lafayette, CO, USA). Lipofectamine 2000 reagent (catalog no. 11668030, Invitrogen) was used to transfect DRGN cultures as described by us previously (*54*). Briefly, the siRNA and transfection reagent were diluted in NBA (without antibiotics) and incubated for 5 min at room temperature before the two solutions were combined, gently mixed, and incubated for a further 25 min at room temperature to form siRNA-reagent complexes. Complexes were diluted to the desired concentrations in NBA, added to the cells, transfected for 5 hours before addition of supplementary NBA to a final volume of 500 μl per well, and incubated at 37°C and 5% CO_2 for 4 days. NBA alone, Lipofectamine alone (Sham), and Lipofectamine + siEGFP (siEGFP) were used as controls. A dose-response assay was undertaken initially, with both siChk1 and siChk2 at 5, 10, 20, 50, and 100 nM concentrations, confirming that a concentration of 10 nM of each optimally knocked down the appropriate mRNA.

Optimal concentrations of each siRNA were then used to determine the effect of Chk1 and Chk2 knockdown on DRGN survival and neurite outgrowth. Immunocytochemistry for βIII-tubulin (catalog no. T4062, Sigma-Aldrich) that marks DRGN soma and neurites was used to quantify survival and neurite outgrowth as described below and by us previously (*52*). All in vitro experiments consisted of three wells per treatment condition and repeated with cultures from at least three independent animals.

SMARTvector Lentiviral rat Chk1 shRNA (shChk1; catalog no. V3SR11242-239228992) and Chk2 shRNA (shChk2; catalog no. V3SR11242-243372901) driven by a cytomegalovirus (CMV) promoter were purchased from Dharmacon. Vectors were grown in the presence of ampicillin, and plasmid DNA was prepared according to the manufacturer's instructions. DRGN cultures were transfected

with appropriate shRNA using in vivo jetPEI (Polyplus Transfection, New York, USA) according to the manufacturer's instructions and as described by us previously (*20*). DRGN were transfected with 0.5, 1, 2, 3, and 4 μg of plasmid DNA containing control empty vector (shNull; CMV promoter but empty vector), shChk, or shChk2. Additional controls included untreated DRGN (NBA) and DRGN transfected with in vivo-jetPEI only (Sham). DRGN were allowed to incubate for 4 days before harvesting of cells and extraction of total RNA for validation of Chk1 and Chk2 mRNA knockdown using qRT-PCR, as described below. Immunocytochemistry for βIII-tubulin that marks DRGN soma and neurites was used to quantify survival and neurite outgrowth as described below and by us previously (*52*). All in vitro experiments consisted of three wells per treatment condition and repeated with cultures from at least three independent animals.

Immunocytochemistry

Cells were fixed in 4% paraformaldehyde (catalog no. F006, TAAB laboratories, Aldermaston, UK) and washed in three changes of phosphate-buffered saline (PBS) before being subjected to immunocytochemistry as described by us previously (*17*, *52*). To visualize neurites, DRGN or RGCs were stained with monoclonal anti–βIII-tubulin antibodies (catalog no. T4026, Sigma-Aldrich) and detected with Alexa-488 anti-mouse secondary antibodies (catalog no. A-21121, Invitrogen). Slides were then viewed with an epi-fluorescence Axioplan 2 microscope, equipped with an AxioCam HRc and running Axiovision Software (all from Carl Zeiss, Hertfordshire, UK). The proportion of DRGN with neurites, the mean neurite length, and the number of surviving βIII-tubulin^{+} RGCs were calculated using Axiovision Software by an investigator masked to the treatment conditions, as previously described (*17*, *52*).

DC crush injury model

Rats were injected subcutaneously with 0.05 ml of buprenorphine to provide analgesia before surgery and anesthetized using 5% of isoflurane in O_2 (1.8 ml/liter) with body temperature and heart rate monitored throughout surgery. After partial T8 laminectomy, DCs were crushed bilaterally using calibrated watchmaker's forceps (*19*) and either vehicle, Chk1i, Chk2i, BML-277, or prexasertib and were injected intrathecally. All inhibitors for in vivo use were prepared in 20% Captisol (catalog no. S4592, Stratech Scientific Ltd., Cambridge, UK) (*42*). The subarachnoid space was cannulated with a polyethylene tube (PE-10; catalog no. 427401, Beckton Dickinson) through the atlanto-occipital membrane as described by others (*55*). The catheter tip was advanced 8 cm caudally to the L1 vertebra, and the other end of the catheter was sealed with a stainless steel plug and affixed to the upper back. Animals were injected immediately with vehicle (PBS) or Chk inhibitors followed by a 10-μl PBS catheter flush. Injections were repeated every 24 hours, and drugs and vehicle reagents were delivered over a 1-min time period using a Hamilton microliter syringe (Hamilton Co., USA).

Chk2 inhibition studies in the DC crush injury model

In pilot dose finding experiments, Chk2 inhibition by Chk2i, BML-277, and prexasertib were all intrathecally injected as described above at 1, 2, 3, 5, and 10 μg ($n = 3$ rats per group, two independent repeats) in a final volume of 10 μl in 20% Captisol (Stratech Scientific Ltd.) either every other day, twice weekly, or once weekly for 28 days (*12*). Rats were then killed, and L4/L5 DRG on both sides were dissected out, pooled together ($n = 4$ DRG per rat; $n = 12$ DRG per group), lysed in ice cold lysis buffer, separated on 12% SDS–polyacrylamide gel electrophoresis (SDS-PAGE) gels, and subjected to Western blot detection of pChk2 levels (*19*). We determined that the amount of Chk2i, BML-277, and prexasertib required to optimally reduce pChk2 levels by intrathecal delivery was 2 μg (final conc., 1.37 mM), 3 μg (final conc., 451.9 μM), and 2 μg (final conc., 364.9 μM), respectively, with an optimal dosing frequency of every 7 days for all Chk2i used in this study. The optimal doses of all Chk2 inhibitors were then used for experiments described in this manuscript. Chk1i (LY2603618) was used at equimolar concentrations for each experiment. Rats were killed in a rising concentration of CO_2 at either 28 days for immunohistochemistry and Western blot analyses or 6 weeks for electrophysiology and functional tests.

The dose de-escalation study was performed using 0.2 μg (final conc., 36.49 μM) and 0.02 μg (final conc., 3.649 μM) of prexasertib and injected intrathecally once every 7 days as described above. For these experiments, $n = 6$ rats per group were used and repeated on two independent occasions (i.e., $n = 12$ rats).

To perform an initial dose response study to knock down Chk2 in vivo after DC injury by shRNA, 1 to 4 μg of plasmid DNA for shNull, shChk1, and shChk2 (all from Dharmacon) were complexed in in vivo JetPEI (catalog no. 101000040, Polyplus, New York, USA) and injected intra-DRG as described by us previously (*20*). Sham-treated animals (partial laminectomy but no DC injury) were also included as additional controls. At 4 weeks after DC injury and treatment, ipsilateral L4/L5 DRG pairs were harvested, total RNA was extracted using TRIzol reagent as described above, and Chk1 and Chk2 mRNA were knocked down using quantitative qRT-PCR, as described above. Contralateral L4/L5 DRG pairs were treated the same as above and used as controls. In further experiments, the optimal dose of 2 μg of each respective shRNA was used. This included Western blot to determine pChk1 and pChk2 levels after shChk2 treatment. For these experiments, animals were randomly assigned to DC + shNull and DC + shChk2 groups, each comprising $n = 6$ rats and repeated on three independent occasions (total, $n = 18$ rats per group). Ipsilateral L4/L5 DRG pairs were harvested at 4 weeks after DC injury and treatment, and total protein was extracted, subjected to Western blot, and probed for pChk1 and pChk2 to determine pChk2 suppression after shChk2-mediated knockdown of Chk2 mRNA.

Last, to determine whether Chk2 suppression by shChk2 also promotes similar levels of electrophysiological, sensory, and locomotor improvements as Chk2i, $n = 6$ rats per group [three independent repeats (total, $n = 18$ rats per group)], animals were randomly assigned to sham, shNull, shChk1, and shChk2 groups. Animals received intra-DRG injections of shNull, shChk1, and shChk2 immediately after DC injury, as described by us previously (*20*). Animals were allowed to survive for 6 weeks with functional testing (tape sensing + removal and ladder crossing tests) performed pre- and post-DC injury as described below. Electrophysiology was performed on the same set of animals at 6 weeks after DC injury and treatment as described below.

ONC injury model

Optic nerves were crushed bilaterally 2 mm from the globe of the eye as described previously (*56*). In pilot dose-finding experiments, Chk2i was intravitreally injected at 1, 2, 3, 5, and 10 μg ($n = 3$ rats per group, two independent repeats), without damaging the lens, immediately after ONC. To determine optimal doses and dosing

frequency, Chk2i was injected every other day, twice weekly, or once every 7 days, in a final volume of 5 μl Captisol (20%) for 24 days. Rats were then killed, and retinae were dissected out, lysed in ice-cold lysis buffer, separated on 12% SDS-PAGE gels, and subjected to Western blot detection of pChk2 levels. We determined that the dosing frequency of twice weekly and 2 μg of Chk2i optimally reduced pChk2 levels. Chk1i was used at the same dose as Chk2i. Optimal doses were then used for all experiments described in this manuscript. Rats were killed in rising concentrations of CO_2 at 24 days after ONC injury for Western blot analyses or for determination of RGC survival and axon regeneration, as described below.

For the experiments reported in this manuscript, $n = 6$ rats per group were used and assigned to (i) intact controls (no surgery to detect baseline parameters); (ii) ONC + vehicle (ONC followed by intravitreal injection of vehicle solution); (iii) ONC + Chk1i (ONC followed by intravitreal injection of equimolar concentration of Chk1i, twice weekly); and (iv) ONC + Chk2i (ONC followed by intravitreal injection of 2 μg of Chk2i). Each experiment was repeated on three independent occasions with a total $n = 18$ rats per group per test.

Assessment of RGC survival

FluoroGold (FG) backfilled RGCs in retinal wholemounts were used to determine RGC survival as described previously (*56*). Briefly, at 22 days after ONC, 2 μl of 4% FG (catalog no. BT80023, Cambridge Bioscience, Cambridge, UK) was injected into the optic nerve (ON), between the lamina cribrosa and the ONC site. Animals were euthanized 2 days later by CO_2 overdose, the retinae were immersion fixed in 4% paraformaldehyde (TAAB Laboratories, Aldermaston, UK), flattened onto charged glass microscope slides, air dried, and mounted in Vectashield mounting medium (catalog no. H-1200, Vector Laboratories, Peterborough, UK). Retinae were randomized and photographed using a Zeiss epi-fluorescence microscope (Zeiss Axioplan 2) equipped with a digital camera (Axiocam HRc) in Axiovision 4 (all from Zeiss, Hertfordshire, UK). The number of FG-labeled RGCs was then counted blind using ImagePro Version 6.0 (Media Cybernetics) from captured images of 12 rectangular areas (0.36 mm by 0.24 mm), 3 from each quadrant, and placed at radial distances from the center of the optic disc of the inner (1/6 eccentricity), midperiphery (1/2 eccentricity), and outer retina (5/6 eccentricity), as described by us previously (*26*). The number of FG-labeled cells in the 12 images was divided by the area of the counting region and pooled together to calculate mean densities of FG-labeled RGC/mm^2 for each retina (*26*).

Immunohistochemistry

Tissue preparation for cryostat sectioning and immunohistochemistry were performed as described by us previously (*19*). Briefly, rats were intracardially perfused with 4% formaldehyde and L4/L5 DRG and segments of T8 cord containing the DC injury sites, and optic nerves were dissected out and postfixed for 2 hours at room temperature. Tissues were then cryoprotected in a sucrose gradient before mounting in optimal cutting temperature (OCT) embedding medium (catalog no. AGR1180, Agar Scientific, Essex, UK) and frozen on dry ice. Samples were then sectioned using a cryostat, and immunohistochemistry was performed on sections from the middle of the DRG or optic nerve as described previously (*17*, *19*). Sections were permeabilized using 0.1% Triton X-100 in PBS, blocked in 3% bovine serum albumin containing 0.05% Tween-20 in PBS, and stained with mouse anti-γH2Ax (1:400 dilution; catalog no. 05-636, Merck Millipore, Watford, UK), rabbit anti-NF200 (1:400 dilution; catalog no. N4142, Sigma-Aldrich, Poole, UK), and mouse anti-GAP43 (1:400 dilution; catalog no. 33-5000, Invitrogen, Poole, UK) primary antibodies overnight at 4°C.

Despite others demonstrating successful Cholera toxin B labeling (*57*), in our hands, it did not label regenerating axons in the rat (*18*, *20*). Hence, we have used GAP43 immunohistochemistry to detect DC axon regeneration, as has been used by us previously (*18*). After washing in PBS, sections were incubated with Alexa-488 anti-mouse and Texas red anti-rabbit immunoglobulin G secondary antibodies for 1 hour at room temperature before further washes in PBS and mounting in Vectashield containing 4′,6-diamidino-2-phenylindole (catalog no. H-1200, Vector Laboratories). Controls were included in each run where the primary antibodies were omitted, and these sections were used to set the background threshold prior to image capture. Sections were viewed using Axioplan 200, an epi-fluorescence microscope equipped with an Axiocam HRc and running Axiovision Software (all from Zeiss, Herefordshire, UK). Image capture and analysis were performed by an investigator masked to the treatment conditions.

Quantification of DC axon regeneration

$GAP43^+$ axons were quantified according to previously published methods. Briefly, the number of intersections of $GAP43^+$ fibers was counted through a dorsoventral orientated line in reconstructed serial parasagittal sections of the cord (serial 50-μm-thick sections, ~70 to 80 sections per animal; $n = 10$ rats per treatment). Axon number was then represented as percentage of fibers counted at 4 mm above the lesion, where the DC was intact.

Quantification of RGC axon regeneration

The number of regenerating $GAP43^+$ axons were counted at ×400 magnification in ON sections after drawing a vertical line through the axons and counting the number of axons extending beyond this line, using previously published methods (*58*). Briefly, an observer, blinded to the identity of each sample, counted the number of $GAP43^+$ axons at 0.2, 0.5, 1.0, 1.5, 2.0, 3.0, and 4.0 mm distal to the lesion site in four longitudinal sections of each nerve ($n = 9$ rats/18 ON/treatment). The diameter of the nerve at each counting distance was also measured using Axiovision Software (Zeiss), and the number of axons per millimeter of nerve width was calculated and averaged over the sections and the total number of axons ($\cdot a_d$) extending distances d, in an ON of radius r estimated by summing over all sections with a thickness (t) of 15 μm using the following formula

$$\sum a_d = \pi r^2 x \text{ (average axons mm}^{-1}\text{)}$$

Protein extraction and Western blot analysis

Total protein from ipsilateral L4/L5 DRG was extracted and subjected to Western blot followed by densitometry according to our previously published methods (*17*, *52*). Briefly, 40 μg of total protein extract was resolved on 12% SDS gels, transferred to polyvinylidene fluoride (PVDF) membranes (catalog no. IPVH00010, Millipore, Watford, UK), and probed with relevant primary antibodies: anti-pChk1 (catalog no. 2348)/pChk2 (catalog no. 2197) (both used at 1:200 dilution; Cell Signaling Technology, Danvers, CA, USA). Monoclonal β-actin (1:1000 dilution; catalog no. A5441, Sigma-Aldrich) was used as a loading control. Membranes were then

incubated with relevant horseradish peroxidase–labeled secondary antibodies, and bands were detected using an enhanced chemiluminescence kit (catalog no. RPN2236, GE Healthcare, Buckinghamshire, UK). For densitometry, Western blots were scanned into Adobe Photoshop (Adobe Systems Inc., San Jose, CA, USA) and analyzed using the built-in-macros for gel analysis in ImageJ (NIH, USA, http://imagej.nih.gov/ij).

Electroretinography
ERG was recorded (HMsERG, Ocuscience, Kansas City, MO, USA) at 24 days after injury and in uninjured controls and was interpreted using ERG View (Ocuscience) (*59*). Briefly, animals were dark adapted (scotopic) overnight, and flash ERG was recorded from −2.5 to +1 log units with respect to standard flash in half-log unit steps, and photopic (light-adapted) flash ERG was recorded with background illumination of 30,000 mcd/m^2 over the same range. ERG traces were analyzed using ERG View (Ocuscience), and marker position was manually verified and adjusted where necessary by an observer masked to the treatment conditions.

Electrophysiology
Six weeks after surgery or treatment, CAPs were recorded after vehicle, Ck2i, Chk1i, BML-277, and prexasertib treatment as previously described (*20*). Briefly, with the experimenter masked to the treatment conditions, silver wire electrodes applied single-current pulses (0.05 ms) through a stimulus isolation unit in increments (0.2, 0.3, 0.6, 0.8, and 1.2 mA) at L1 to L2, and CAPs were recorded at C4 to C5 along the surface of the midline spinal cord. Spike 2 software was then used to calculate CAP amplitudes between the negative deflection after the stimulus artifact and the next peak of the wave. CAP area was calculated by rectifying the negative CAP component (full-wave rectification) and measuring its area. At the different stimulation intensities, the dorsal half of the spinal cord was transected between the stimulating and recording electrodes at the end of the experiment to confirm that a CAP could not be detected. Representative CAP traces are processed output data from Spike 2 software.

Functional tests
Functional testing after DC lesions was carried out as described previously (*12*). Briefly, animals (*n* = 6 rats per group, three independent repeats; total, *n* = 18 per group) received training to master traversing the horizontal ladder for 1 week before functional testing. Baseline parameters for all functional tests were established 2 to 3 days before injury. Animals were then tested 2 days after DC lesion + treatment and then weekly for 6 weeks. Experiments were performed by two observers (treatment conditions were masked) in the same order, the same time of day, and each test performed for three individual trials.

Horizontal ladder test
This tests the animals' locomotor function and is performed on a 0.9-m-long horizontal ladder with a diameter of 15.5 cm and randomly adjusted rungs with variable gaps of 3.5 to 5.0 cm. The total number of steps taken to cross the ladder and the left and right rear paw slips were recorded, and the mean error rate was then calculated by dividing the number of slips by the total number of steps taken.

Tape sensing and removal test (sensory function)
The tape sensing and removal test determines touch perception from the left hind paw. Animals were held with both hind paws extended, and the time it took for the animal to detect and remove a 15 mm by 15 mm piece of tape (Kip Hochkrepp, Bocholt, Germany) was recorded and used to calculate the mean sensing time.

Statistical analysis
Data are presented as means ± SEM. When data were normally distributed, significant differences were calculated using Statistical Package for the Social Sciences (SPSS) Version 22 (IBM, NJ, USA) software by one-way ANOVA, with Bonferroni post hoc tests, set at $P < 0.05$.

For the horizontal ladder crossing functional tests, data were analyzed using R package (www.r-project.org), and whole time course of lesioned and sham-treated animals were compared using binomial generalized linear mixed models (GLMMs) as described previously (*12*). Thus, data were compared using binomial GLMMs, with lesioned/sham ("LESION"; set to true in lesioned animals' after surgery, false otherwise) and operated/unoperated ("OPERATED"; set to false before surgery, true after surgery) as fixed factors, animals as a random factor, and time as a continuous covariate. Binomial GLMMs were then fitted in R using package *lme4* with the *glmer* functions, and *P* values were calculated using parametric bootstrap.

For the tape sensing and removal test, linear mixed models were calculated by model comparison in R using the package *pbkrtest*, with the Kenward-Roger method (*20*). Independent sample *t* tests were performed to determine statistical differences at individual time points.

SUPPLEMENTARY MATERIALS
Supplementary material for this article is available at https://science.org/doi/10.1126/sciadv.abq2611

View/request a protocol for this paper from *Bio-protocol*.

REFERENCES AND NOTES
1. K. Aziz, S. Nowsheen, G. Pantelias, G. Iliakis, V. G. Gorgoulis, A. G. Georgakilas, Targeting DNA damage and repair: Embracing the pharmacological era for successful cancer therapy. *Pharmacol. Ther.* **133**, 334–350 (2012).
2. C. Brochier, B. Langley, Chromatin modifications associated with DNA double-strand breaks repair as potential targets for neurological diseases. *Neurotherapeutics* **10**, 817–830 (2013).
3. B. J. Lamarche, N. I. Orazio, M. D. Weitzman, The MRN complex in double-strand break repair and telomere maintenance. *FEBS Lett.* **584**, 3682–3695 (2010).
4. J. H. Lee, T. T. Paull, ATM activation by DNA double-strand breaks through the Mre11-Rad50-Nbs1 complex. *Science* **308**, 551–554 (2005).
5. Y. Shiloh, Y. Ziv, The ATM protein kinase: Regulating the cellular response to genotoxic stress, and more. *Nat. Rev. Mol. Cell Biol.* **14**, 197–210 (2013).
6. A. N. Blackford, S. P. Jackson, ATM, ATR, and DNA-PK: The trinity at the heart of the DNA damage response. *Mol. Cell* **66**, 801–817 (2017).
7. R. R. Kotipatruni, V. R. Dasari, K. K. Veeravalli, D. H. Dinh, D. Fassett, J. S. Rao, p53- and Bax-mediated apoptosis in injured rat spinal cord. *Neurochem. Res.* **36**, 2063–2074 (2011).
8. T. Hayashi, M. Sakurai, K. Abe, M. Sadahiro, K. Tabayashi, Y. Itoyama, Apoptosis of motor neurons with induction of caspases in the spinal cord after ischemia. *Stroke* **29**, 1007–1013 (1998).
9. A. McShea, P. L. Harris, K. R. Webster, A. F. Wahl, M. A. Smith, Abnormal expression of the cell cycle regulators P16 and CDK4 in Alzheimer's disease. *Am. J. Pathol.* **150**, 1933–1939 (1997).
10. Z. Nagy, M. M. Esiri, A. M. Cato, A. D. Smith, Cell cycle markers in the hippocampus in Alzheimer's disease. *Acta Neuropathol.* **94**, 6–15 (1997).
11. E. Fielder, T. von Zglinicki, D. Jurk, The DNA damage response in neurons: Die by apoptosis or survive in a senescence-like state? *J. Alzheimers Dis.* **60**, S107–S131 (2017).
12. R. I. T. Tuxworth, M. J. Taylor, A. M. Anduaga, A. Hussien-Ali, S. Chatzimatthaiou, J. Longland, A. M. Thompson, S. Almutiri, P. Alifragis, C. P. Kyriacou, B. Kysela, Z. Ahmed, Attenuating the DNA damage response to double-strand breaks restores function in models of CNS neurodegeneration. *Brain Commun.* **1**, fcz005 (2019).
13. M. J. Taylor, R. I. Tuxworth, Continuous tracking of startled *Drosophila* as an alternative to the negative geotaxis climbing assay. *J. Neurogenet.* **33**, 190–198 (2019).
14. S. Nandakumar, O. Grushko, L. A. Buttitta, Polyploidy in the adult *Drosophila* brain. *eLife* **9**, e54385 (2020).

15. Y. Sanchez, C. Wong, R. S. Thoma, R. Richman, Z. Wu, H. Piwnica-Worms, S. J. Elledge, Conservation of the Chk1 checkpoint pathway in mammals: Linkage of DNA damage to Cdk regulation through Cdc25. *Science* **277**, 1497–1501 (1997).
16. M. H. Brodsky, B. T. Weinert, G. Tsang, Y. S. Rong, N. M. McGinnis, K. G. Golic, D. C. Rio, G. M. Rubin, Drosophila melanogaster MNK/Chk2 and p53 regulate multiple DNA repair and apoptotic pathways following DNA damage. *Mol. Cell. Biol.* **24**, 1219–1231 (2004).
17. Z. Ahmed, E. L. Suggate, E. R. Brown, R. G. Dent, S. J. Armstrong, L. B. Barrett, M. Berry, A. Logan, Schwann cell-derived factor-induced modulation of the NgR/p75NTR/EGFR axis disinhibits axon growth through CNS myelin in vivo and in vitro. *Brain* **129**, 1517–1533 (2006).
18. Z. Ahmed, D. Bansal, K. Tizzard, S. Surey, M. Esmaeili, A. M. Gonzalez, M. Berry, A. Logan, Decorin blocks scarring and cystic cavitation in acute and induces scar dissolution in chronic spinal cord wounds. *Neurobiol. Dis.* **64**, 163–176 (2014).
19. S. Surey, M. Berry, A. Logan, R. Bicknell, Z. Ahmed, Differential cavitation, angiogenesis and wound-healing responses in injured mouse and rat spinal cords. *Neuroscience* **275**, 62–80 (2014).
20. S. Almutiri, M. Berry, A. Logan, Z. Ahmed, Non-viral-mediated suppression of AMIGO3 promotes disinhibited NT3-mediated regeneration of spinal cord dorsal column axons. *Sci. Rep.* **8**, 10707 (2018).
21. C. B. McGuire, G. J. Snipes, J. J. Norden, Light-microscopic immunolocalization of the growth- and plasticity-associated protein GAP-43 in the developing rat brain. *Brain Res.* **469**, 277–291 (1988).
22. F. Widmer, P. Caroni, Identification, localization, and primary structure of CAP-23, a particle-bound cytosolic protein of early development. *J. Cell Biol.* **111**, 3035–3047 (1990).
23. D. Chung, A. Shum, G. Caraveo, GAP-43 and BASP1 in axon regeneration: Implications for the treatment of neurodegenerative diseases. *Front. Cell Dev. Biol.* **8**, 567537 (2020).
24. J. M. Lee, J. Nair, A. Zimmer, S. Lipkowitz, C. M. Annunziata, M. J. Merino, E. M. Swisher, M. I. Harrell, J. B. Trepel, M. J. Lee, M. H. Bagheri, D. A. Botesteanu, S. M. Steinberg, L. Minasian, I. Ekwede, E. C. Kohn, Prexasertib, a cell cycle checkpoint kinase 1 and 2 inhibitor, in BRCA wild-type recurrent high-grade serous ovarian cancer: A first-in-class proof-of-concept phase 2 study. *Lancet Oncol.* **19**, 207–215 (2018).
25. V. Vigneswara, M. Berry, A. Logan, Z. Ahmed, Caspase-2 is upregulated after sciatic nerve transection and its inhibition protects dorsal root ganglion neurons from apoptosis after serum withdrawal. *PLOS ONE* **8**, e57861 (2013).
26. Z. Ahmed, H. Kalinski, M. Berry, M. Almasieh, H. Ashush, N. Slager, A. Brafman, I. Spivak, N. Prasad, I. Mett, E. Shalom, E. Alpert, A. Di Polo, E. Feinstein, A. Logan, Ocular neuroprotection by siRNA targeting caspase-2. *Cell Death Dis.* **2**, e173 (2011).
27. V. Vigneswara, N. Akpan, M. Berry, A. Logan, C. M. Troy, Z. Ahmed, Combined suppression of CASP2 and CASP6 protects retinal ganglion cells from apoptosis and promotes axon regeneration through CNTF-mediated JAK/STAT signalling. *Brain* **137**, 1656–1675 (2014).
28. Z. Nagy, L. Czirjak, Predictors of survival in 171 patients with systemic sclerosis (scleroderma). *Clin. Rheumatol.* **16**, 454–460 (1997).
29. N. He, W. L. Jin, K. H. Lok, Y. Wang, M. Yin, Z. J. Wang, Amyloid-β1–42 oligomer accelerates senescence in adult hippocampal neural stem/progenitor cells via formylpeptide receptor 2. *Cell Death Dis.* **4**, e924 (2013).
30. V. Vigneswara, Z. Ahmed, Long-term neuroprotection of retinal ganglion cells by inhibiting caspase-2. *Cell Death Discov.* **2**, 16044 (2016).
31. J. Smith, L. M. Tho, N. Xu, D. A. Gillespie, The ATM-Chk2 and ATR-Chk1 pathways in DNA damage signaling and cancer. *Adv. Cancer Res.* **108**, 73–112 (2010).
32. H. L. Smith, H. Southgate, D. A. Tweddle, N. J. Curtin, DNA damage checkpoint kinases in cancer. *Expert Rev. Mol. Med.* **22**, e2 (2020).
33. I. I. Kruman, E. I. Schwartz, DNA damage response and neuroprotection. *Front. Biosci.* **13**, 2504–2515 (2008).
34. X.-H. Lu, V. B. Mattis, N. Wang, I. Al-Ramahi, N. van den Berg, S. A. Fratantoni, H. Waldvogel, E. Greiner, A. Osmand, K. Elzein, J. Xiao, S. Dijkstra, R. de Pril, H. V. Vinters, R. Faull, E. Signer, S. Kwak, J. J. Marugan, J. Botas, D. F. Fischer, C. N. Svendsen, I. Munoz-Sanjuan, X. W. Yang, Targeting ATM ameliorates mutant Huntingtin toxicity in cell and animal models of Huntington's disease. *Sci. Transl. Med.* **6**, 268ra178 (2014).
35. M. G. Fehlings, N. Theodore, J. Harrop, G. Maurais, C. Kuntz, C. I. Shaffrey, B. K. Kwon, J. Chapman, A. Yee, A. Tighe, L. McKerracher, A phase I/IIa clinical trial of a recombinant Rho protein antagonist in acute spinal cord injury. *J. Neurotrauma* **28**, 787–796 (2011).
36. K. Kucher, D. Johns, D. Maier, R. Abel, A. Badke, H. Baron, R. Thietje, S. Casha, R. Meindl, B. Gomez-Mancilla, C. Pfister, R. Rupp, N. Weidner, A. Mir, M. E. Schwab, A. Curt, First-in-man intrathecal application of neurite growth-promoting anti-Nogo-A antibodies in acute spinal cord injury. *Neurorehabil. Neural Repair* **32**, 578–589 (2018).
37. L. L. Liau, Q. H. Looi, W. C. Chia, T. Subramaniam, M. H. Ng, J. X. Law, Treatment of spinal cord injury with mesenchymal stem cells. *Cell Biosci.* **10**, 112 (2020).
38. Z. Ahmed, M. Aslam, B. Lorber, E. L. Suggate, M. Berry, A. Logan, Optic nerve and vitreal inflammation are both RGC neuroprotective but only the latter is RGC axogenic. *Neurobiol. Dis.* **37**, 441–454 (2010).
39. G. A. Metz, A. Curt, H. van de Meent, I. Klusman, M. E. Schwab, V. Dietz, Validation of the weight-drop contusion model in rats: A comparative study of human spinal cord injury. *J. Neurotrauma* **17**, 1–17 (2000).
40. K. Brechtel, A. Tura, M. Abdibzadeh, S. Hirsch, S. Conrad, J. M. Schwab, Intrinsic locomotor outcome in dorsal transection of rat spinal cord: Predictive value of minimal incision depth. *Spinal Cord* **44**, 605–613 (2006).
41. B. K. Kwon, T. R. Oxland, W. Tetzlaff, Animal models used in spinal cord regeneration research. *Spine (Phila Pa 1976)* **27**, 1504–1510 (2002).
42. C. D. Lowery, A. B. VanWye, M. Dowless, W. Blosser, B. L. Falcon, J. Stewart, J. Stephens, R. P. Beckmann, A. Bence Lin, L. F. Stancato, The checkpoint kinase 1 inhibitor prexasertib induces regression of preclinical models of human neuroblastoma. *Clin. Cancer Res.* **23**, 4354–4363 (2017).
43. O. Campagne, A. Davis, A. R. Maharaj, B. Zhong, J. Stripay, D. Farmer, M. F. Roussel, C. F. Stewart, CNS penetration and pharmacodynamics of the CHK1 inhibitor prexasertib in a mouse group 3 medulloblastoma model. *Eur. J. Pharm. Sci.* **142**, 105106 (2020).
44. E. J. Lampert, A. Cimino-Mathews, J. S. Lee, J. Nair, M. J. Lee, A. Yuno, D. An, J. B. Trepel, E. Ruppin, J. M. Lee, Clinical outcomes of prexasertib monotherapy in recurrent BRCA wild-type high-grade serous ovarian cancer involve innate and adaptive immune responses. *J. Immunother. Cancer* **8**, e000516 (2020).
45. M. M. Bottros, P. J. Christo, Current perspectives on intrathecal drug delivery. *J. Pain Res.* **7**, 615–626 (2014).
46. A. Logan, Z. Ahmed, A. Baird, A. M. Gonzalez, M. Berry, Neurotrophic factor synergy is required for neuronal survival and disinhibited axon regeneration after CNS injury. *Brain* **129**, 490–502 (2006).
47. S. Leon, Y. Yin, J. Nguyen, N. Irwin, L. I. Benowitz, Lens injury stimulates axon regeneration in the mature rat optic nerve. *J. Neurosci.* **20**, 4615–4626 (2000).
48. Y. Yin, M. T. Henzl, B. Lorber, T. Nakazawa, T. T. Thomas, F. Jiang, R. Langer, L. I. Benowitz, Oncomodulin is a macrophage-derived signal for axon regeneration in retinal ganglion cells. *Nat. Neurosci.* **9**, 843–852 (2006).
49. A. Thompson, M. Berry, A. Logan, Z. Ahmed, Activation of the BMP4/Smad1 pathway promotes retinal ganglion cell survival and axon regeneration. *Invest. Ophthalmol. Vis. Sci.* **60**, 1748–1759 (2019).
50. E. Speretta, T. R. Jahn, G. G. Tartaglia, G. Favrin, T. P. Barros, S. Imarisio, D. A. Lomas, L. M. Luheshi, D. C. Crowther, C. M. Dobson, Expression in drosophila of tandem amyloid β peptides provides insights into links between aggregation and neurotoxicity. *J. Biol. Chem.* **287**, 20748–20754 (2012).
51. Z. Ahmed, R. G. Dent, E. L. Suggate, L. B. Barrett, R. J. Seabright, M. Berry, A. Logan, Disinhibition of neurotrophin-induced dorsal root ganglion cell neurite outgrowth on CNS myelin by siRNA-mediated knockdown of NgR, p75NTR and Rho-A. *Mol. Cell. Neurosci.* **28**, 509–523 (2005).
52. Z. Ahmed, R. G. Dent, W. E. Leadbeater, C. Smith, M. Berry, A. Logan, Matrix metalloproteases: Degradation of the inhibitory environment of the transected optic nerve and the scar by regenerating axons. *Mol. Cell. Neurosci.* **28**, 64–78 (2005).
53. F.-Z. Wang, H.-R. Fei, Y.-J. Cui, Y.-K. Sun, Z.-M. Li, X.-Y. Wang, X.-Y. Yang, J.-G. Zhang, B.-L. Sun, The checkpoint 1 kinase inhibitor LY2603618 induces cell cycle arrest, DNA damage response and autophagy in cancer cells. *Apoptosis* **19**, 1389–1398 (2014).
54. P. J. Morgan-Warren, J. O'Neill, F. de Cogan, I. Spivak, H. Ashush, H. Kalinski, Z. Ahmed, M. Berry, E. Feinstein, R. A. H. Scott, A. Logan, siRNA-mediated knockdown of the mTOR inhibitor RTP801 promotes retinal ganglion cell survival and axon elongation by direct and indirect mechanisms. *Invest. Ophthalmol. Vis. Sci.* **57**, 429–443 (2016).
55. T. L. Yaksh, T. A. Rudy, Chronic catheterization of the spinal subarachnoid space. *Physiol. Behav.* **17**, 1031–1036 (1976).
56. M. Berry, J. Carlile, A. Hunter, Peripheral nerve explants grafted into the vitreous body of the eye promote the regeneration of retinal ganglion cell axons severed in the optic nerve. *J. Neurocytol.* **25**, 147–170 (1996).
57. S. Neumann, F. Bradke, M. Tessier-Lavigne, A. I. Basbaum, Regeneration of sensory axons within the injured spinal cord induced by intraganglionic cAMP elevation. *Neuron* **34**, 885–893 (2002).
58. V. Vigneswara, M. Berry, A. Logan, Z. Ahmed, Pigment epithelium-derived factor is retinal ganglion cell neuroprotective and axogenic after optic nerve crush injury. *Invest. Ophthalmol. Vis. Sci.* **54**, 2624–2633 (2013).
59. R. J. Blanch, Z. Ahmed, A. Sik, D. R. J. Snead, P. A. Good, J. O'Neill, M. Berry, R. A. H. Scott, A. Logan, Neuroretinal cell death in a murine model of closed globe injury: Pathological and functional characterization. *Invest. Ophthalmol. Vis. Sci.* **53**, 7220–7226 (2012).

Acknowledgments: We thank the staff at the University of Birmingham's Biomedical Services Unit for assistance with animal studies. **Funding:** This work was supported by UK Biotechnology and Biological Sciences Research Council New Investigator award BB/N008472/1 (R.I.T.), Medical Research Council Confidence in Concept award (Z.A. and R.I.T.), UK Biotechnology and Biological Sciences Research Council–funded Midlands Integrative

Biosciences Training Partnership studentship (M.J.T.), Saudi Education Ministry PhD Studentship (S.H. and Z.A.), and University of Birmingham Bryant Bequest PhD Studentship (A.M.T. and Z.A.). **Author contributions:** Conceptualization: R.I.T and Z.A. Methodology: M.J.T., A.M.T., and S.A. Investigation: M.J.T., A.M.T., and S.A. Visualization: M.J.T., A.M.T., S.A., R.I.T., and Z.A. Supervision: R.I.T. and Z.A. Writing (original draft): M.J.T., R.I.T., and Z.A. Writing (review and editing): R.I.T. and Z.A. **Competing interests:** R.I.T. and Z.A. are inventors on patents related to this work filed by the University of Birmingham (nos. WO 2022/084201 and WO 2022/084202, filed 21 October 2020, published 28 April 2022). The authors declare that they have no other competing interests. **Data and materials availability:** All data needed to evaluate the conclusions in the paper are present in the paper and/or the Supplementary Materials. Additional data related to this paper may be requested from the authors.

Submitted 28 March 2022
Accepted 29 July 2022
Published 14 September 2022
10.1126/sciadv.abq2611
