# Supplementary material for: Inhibition of Chk2 promotes neuroprotection, axon regeneration, and functional recovery after CNS injury
Source: Sci Adv. 2022 Sep 14;8(37):eabq2611. doi: 10.1126/sciadv.abq2611 (PMC9473583; doi:10.1126/sciadv.abq2611)
Supplement: Supplementary file 2 — Figs. S1 to S7 [file sciadv.abq2611_sm.pdf]

Science Advances
AAAS

Supplementary Materials for

Inhibition of Chk2 promotes neuroprotection, axon regeneration, and functional recovery after CNS injury

Matthew J. Taylor *et al.*

Corresponding author: Richard I. Tuxworth, r.i.tuxworth@bham.ac.uk; Zubair Ahmed, z.ahmed.1@bham.ac.uk

Sci. Adv. **8**, eabq2611 (2022)
DOI: 10.1126/sciadv.abq2611

The PDF file includes:

Figs. S1 to S7
Legends for data S1 and S2

Other Supplementary Material for this manuscript includes the following:

Data S1 and S2

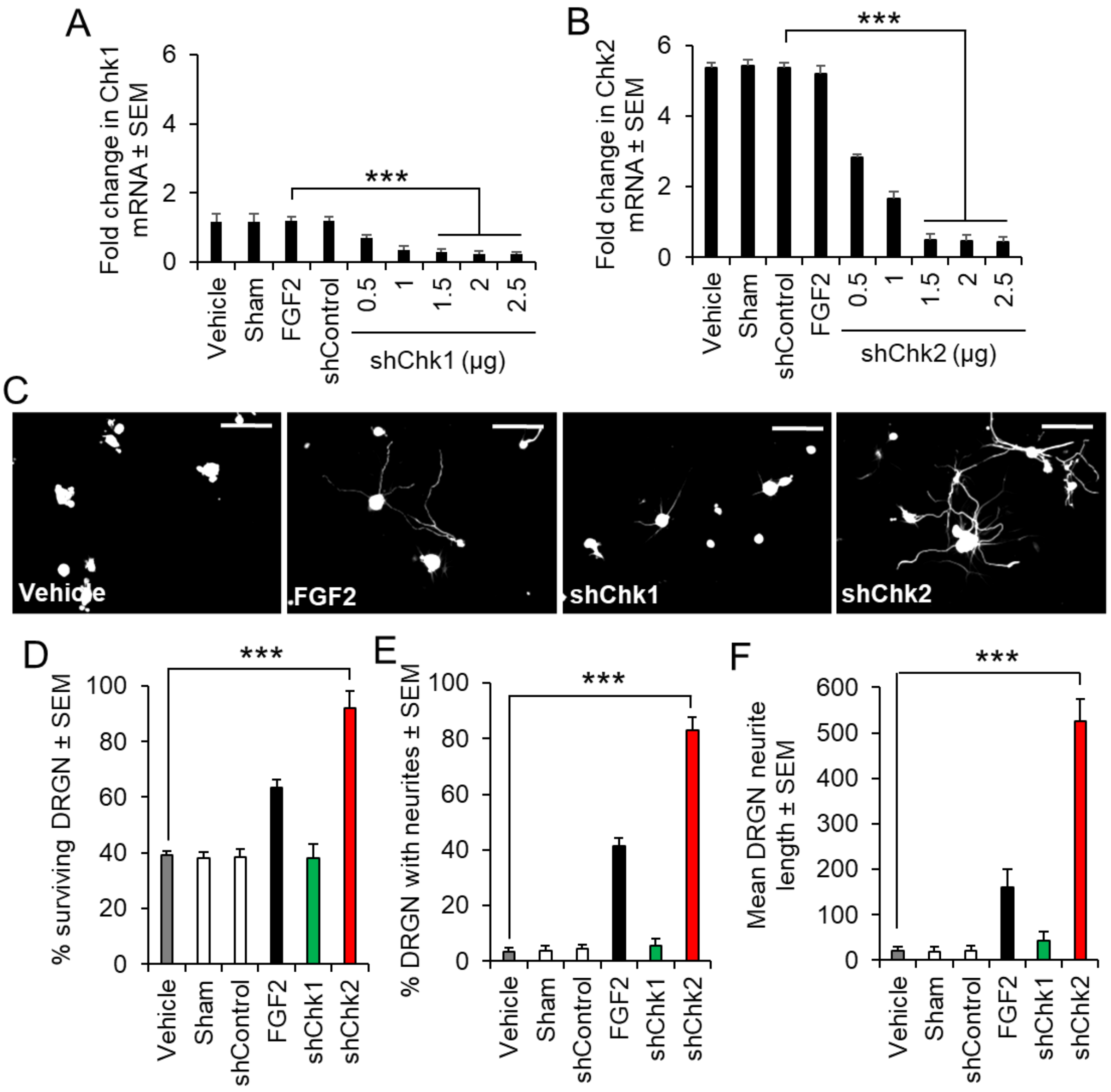

Fig. S1.

Knockdown of Chk1 and Chk2 in DRGN cultures after 4 days using PEI delivered plasmid DNA encoding shChk1 and shCk2. Fold change in mRNA after (**A**) shChk1 and (**B**) shChk2 treatment, respectively, showing optimal knockdown of both Chk1 and Chk2, respectively with 1.5µg of each respective plasmid DNA. (**C**) Representative images from Sham, FGF2 (positive control), 1.5µg of shChk1 and 1.5µg of shChk2 plasmid DNA and demonstrating significantly increased (**D**) % surviving DRGN, (**E**) % DRGN with neurites and (**F**) mean DRGN neurite length compared to control NBA, Sham non-specific shEGFP and positive control FGF2 treatment. *n* = 3 wells/treatment/test, 3 independent repeats (total *n* = 9 wells/treatment/test). *** = P<0.0001, ANOVA.

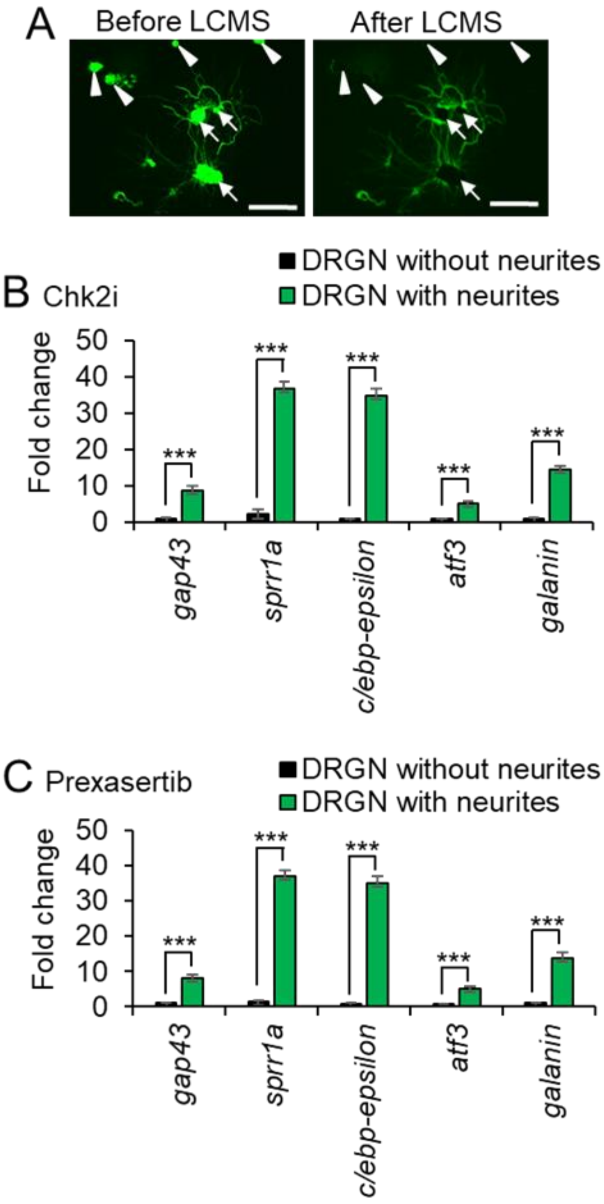

Fig. S2.

Laser capture microdissection (LCM) of DRGN and expression of RAGs after exposure to Chk2i and Prexasertib *in vitro*. (**A**) LCM of DRGN before and after collection of DRGN somata from FMI-43+ DRGN with (arrows) and without neurites (arrowheads). (**B**) Fold change in RAG mRNA expression levels in DRGN with and without neurites after exposure to Chk2i levels (normalized to mRNA levels in DRGN treated with Chk1i). (**C**) Fold change in RAG mRNA expression levels in DRGN with and without neurites after exposure to Chk2i levels (normalized to mRNA levels in DRGN treated with Chk1i). Scale bars in (A) = 100µm. *n* = 1000 DRGN/treatment, 6 independent repeats (total *n* = 6000 DRGN/treatment group). *** = P<0.0001, ANOVA.

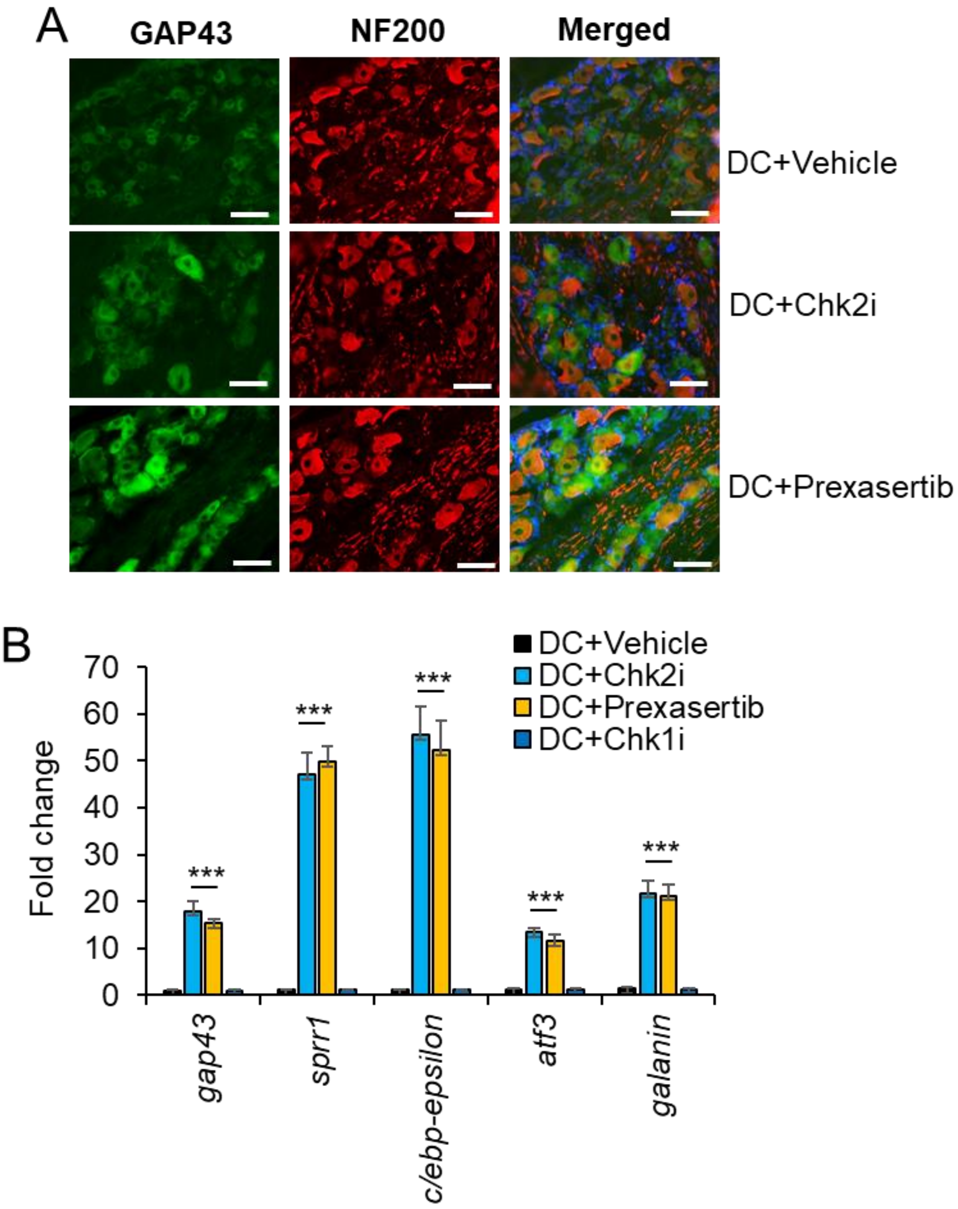

Fig. S3.

GAP43 immunohistochemistry and expression of RAGs in DRG after treatment for 10 days with Chk2 and Prexasertib *in vivo*. (**A**) Immunohistochemistry for GAP43 (green) and NF200 (red) in DRGN at 10 days after DC injury and treatment with Chk2i and Prexasertib. Merged image = GAP43 (green), NF200 (red) and DAPI^{+} nuclei (blue). (**B**) RAG expression after treatment with Chk2i, Prexasertib and Chk1 to show significant upregulation of RAG mRNA levels in DRG after treatment with Chk2i and Prexasertib and little or no change after DC+Vehicle or DC-Chk1i treatment. Scale bars in (A) = 25μm. *n* = 8 rats/treatment. *** = P<0.0001, ANOVA.

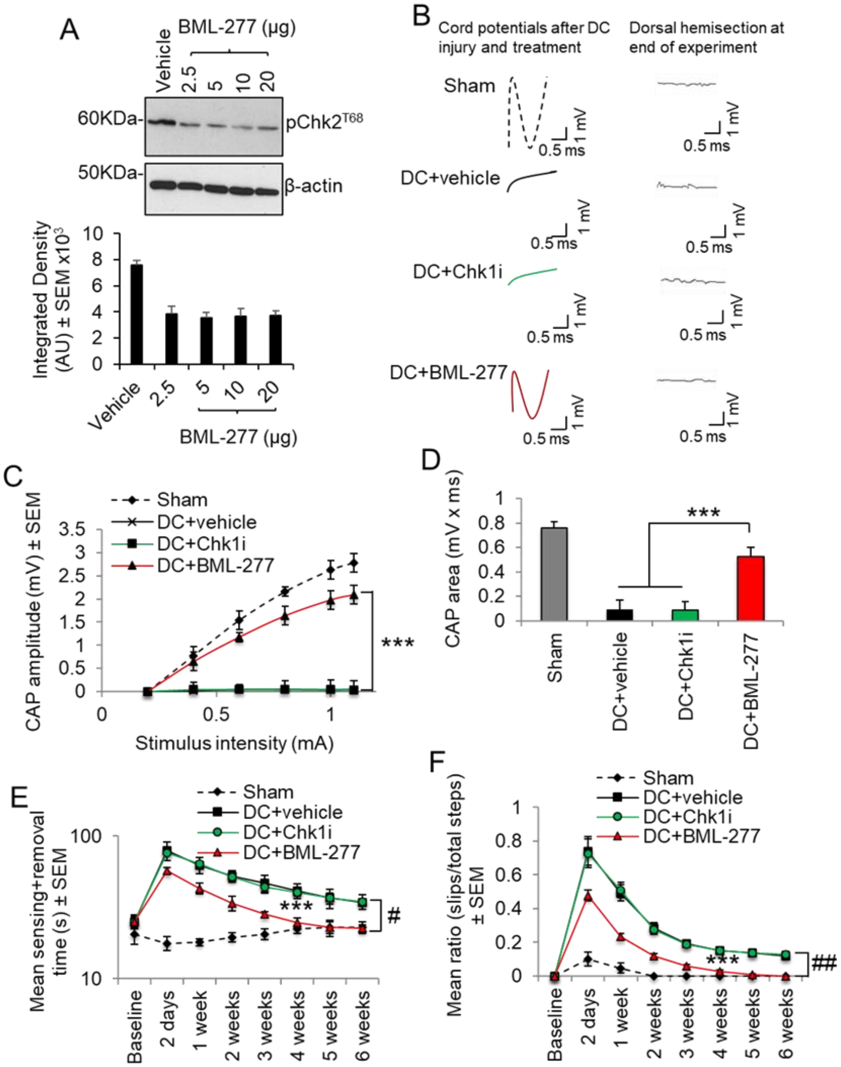

Fig. S4.

Inhibition of Chk2 with BML-277 confirms that suppression of Chk2 and not Chk1 promotes significant functional recovery after DC injury *in vivo*. (**A**) Western blot and densitometry show that BML-277 also significantly suppresses pChk2T68 levels. (**B**) Spike 2 software-processed CAP traces at 6 weeks after DC injury from representative Sham controls, DC+vehicle, DC+shChk1i and DC+BML-277-treated rats. (**C**) Negative CAP amplitudes and (**D**) mean CAP area at different stimulation intensities were significantly attenuated in DC+vehicle- and DC+shChk1-treated rats but were restored in DC+BML-277-treated rats (P<0.0001, one-way ANOVA (main effect)). (**E**) Mean tape sensing and removal times were restored to normal 4 weeks after treatment with BML-277 (P<0.00012, independent sample t-test (DC+vehicle vs. DC+BML-277 at 4 weeks) whilst a significant deficit remained in DC+vehicle- and DC+shChk1-treated rats (# = P<0.00011, generalized linear mixed models over the whole 6 weeks). (**F**) Mean error ratio to show the number of slips vs total number of steps in the horizontal ladder walking test also returns to normal 4 weeks after treatment with BML-277 (P<0.00012, independent sample t-test (DC+vehicle vs DC+BML-277 at 4 weeks)), with a deficit remaining in DC+vehicle- and DC+shChk1-treated rats (## = P<0.00011, linear mixed models over the whole 6 weeks). *n* = 6 rats/treatment/test, 3 independent repeats (total *n* = 18 rats/treatment/test).

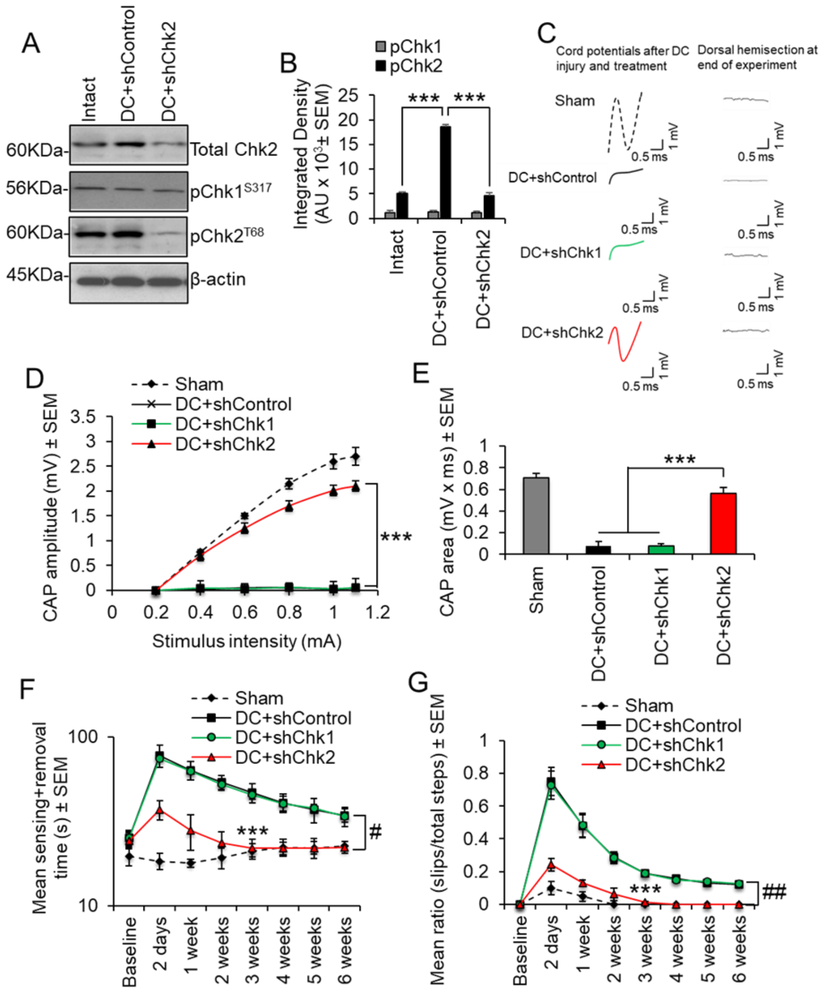

Fig S5.

Inhibition of Chk2 using non-viral *in vivo*-JetPEI (PEI) promotes significant functional repair after DC injury *in vivo*. (A) Western blot and subsequent (B) densitometry after treatment with PEI delivered plasmids significantly suppressed pChk2 levels in spinal L4/L5 DRGs at 4 weeks after DC injury without affecting pChk1 levels. *n* = 12 DRG/treatment (6 rats/treatment), 3 independent repeats (total *n* = 36 DRG/treatment (18 rats/treatment)). (**C**) Spike 2 software-processed CAP traces at 6 weeks after DC injury from representative Sham controls, DC+shNull, DC+shChk1 and DC+shChk2-treated rats. (**D**) Negative CAP amplitudes and (**E**) CAP area was significantly attenuated in DC+shNull- and DC+shChk1-treated rats but were restored in DC+shChk2-treated rats (P<0.0001, one-way ANOVA (main effect)). (**F**) Mean tape sensing and removal times were restored to normal 3 weeks after treatment with shChk2 (P<0.0001, independent sample t-test (DC+shNull vs. DC+shChk2 at 3 weeks) whilst a significant deficit remained in DC+shNull- and DC+shChk1-treated rats (# = P<0.00013, generalized linear mixed models over the whole 6 weeks). (**G**) Mean error ratio to show the number of slips vs total number of steps in the horizontal ladder walking test also returns to normal 3 weeks after treatment with shChk2 (P<0.00011, independent sample t-test (DC+shNull vs DC+shChk2 at 3 weeks)), with a deficit remaining in DC+shNull- and DC+shChk1-treated rats (## = P<0.0001, linear mixed models over the whole 6 weeks). *n* = 6 rats/treatment/test, 3 independent repeats (total *n* = 18 rats/treatment/test).

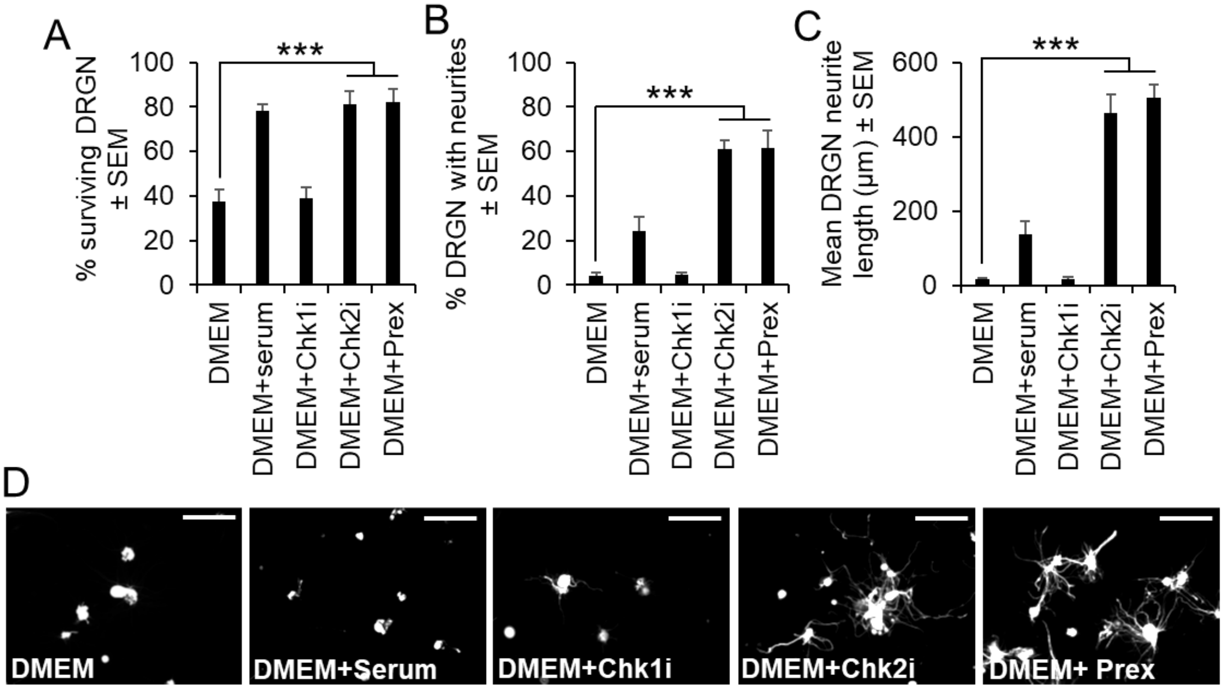

Fig S6.

Chk2 inhibition is DRGN neuroprotective in an *in vitro* serum withdrawal model. Chk2i and Prexasertib (Prex) promotes significant (**A**) DRGN survival, increases the (**B**) number of DRGN with neurites and the (**C**) mean neurite length. ***P<0.0001, ANOVA with Dunnett's post-hoc test. (**D**) Representative images to show DRGN survival and neurite outgrowth after Chk2 inhibition. Scale bars in (D) = 100µm. *n* = 3 wells/treatment, 3 independent repeats (total *n* = 9 wells/treatment).

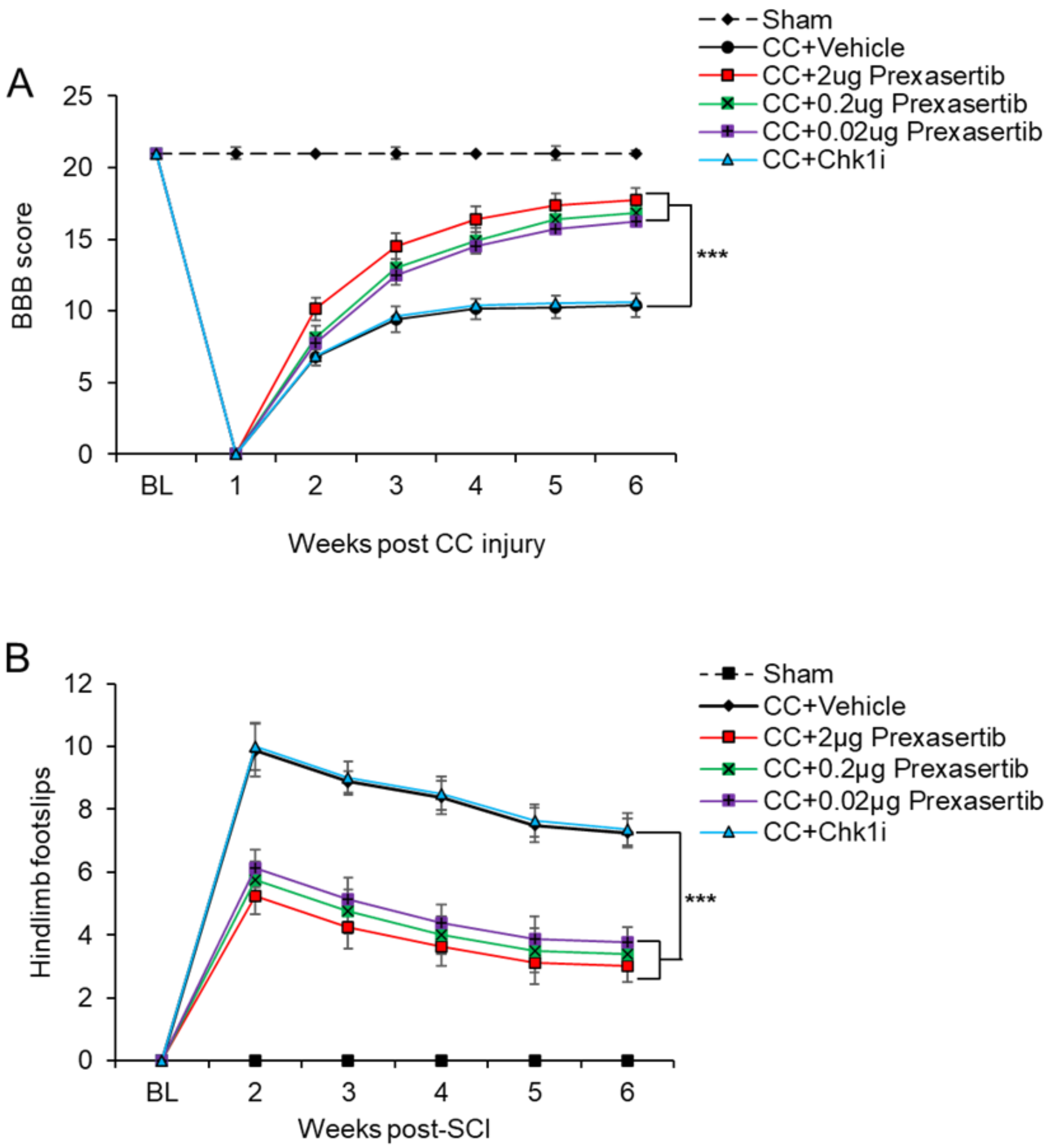

Fig S7.
Chk2 inhibition promotes functional recovery in a severe clip compression (CC) injury model of SCI. is DRGN neuroprotective in an *in vitro* serum withdrawal model. (**A**) BBB and (**B**) ladder crossing performance both significantly increases at all doses of Prexasertib compared to CC+Vehicle or CC+Chk1i-treated rats. ***P<0.0001, ANOVA with Dunnett's post-hoc test. *n* = 12 rats/treatment wells/treatment.

Data S1. (separate file)
Source Data.

Data S2. (separate file)
Original full length Western blots
