# Supplementary material for: Inhibition of Chk2 promotes neuroprotection, axon regeneration, and functional recovery after CNS injury
Source: Sci Adv. 2022 Sep 14;8(37):eabq2611. doi: 10.1126/sciadv.abq2611 (PMC9473583; doi:10.1126/sciadv.abq2611)
Supplement: Supplementary file 3 — Data S1 and S2 [file sciadv.abq2611_data_s1_and_s2.zip › sciadv.abq2611_data_s2.pdf]

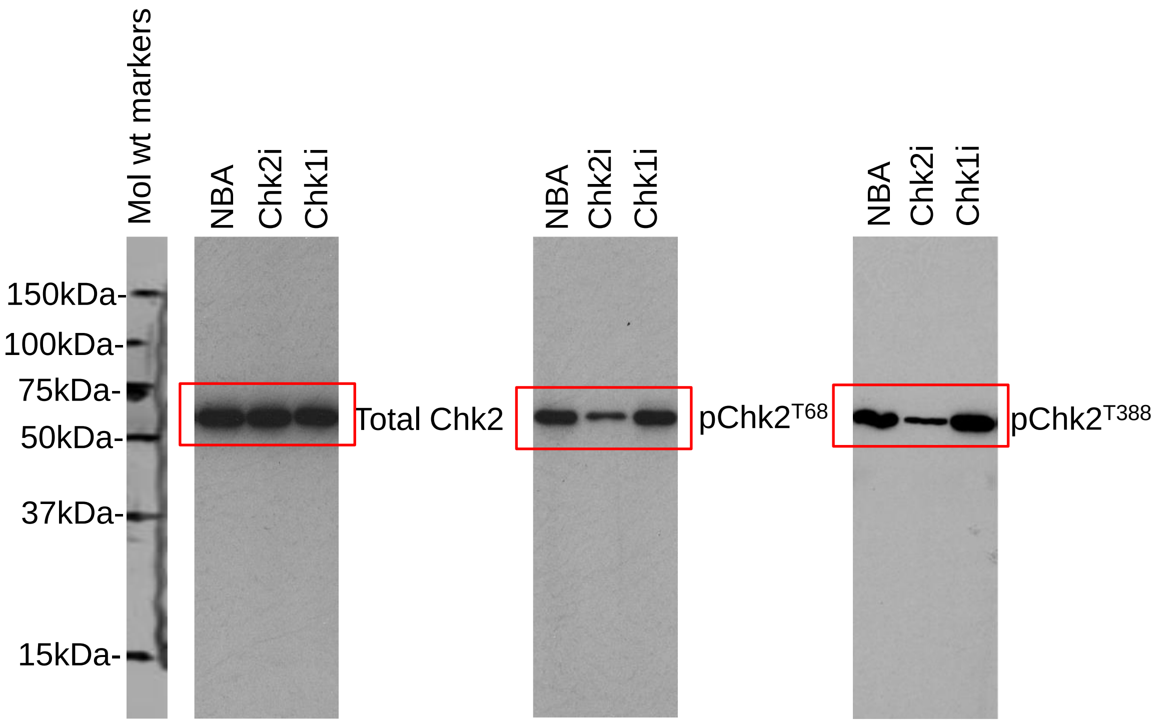

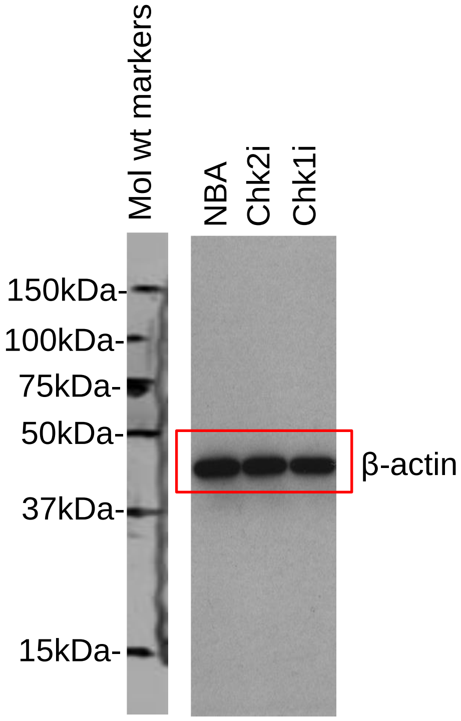

Fig. 2A Original blots

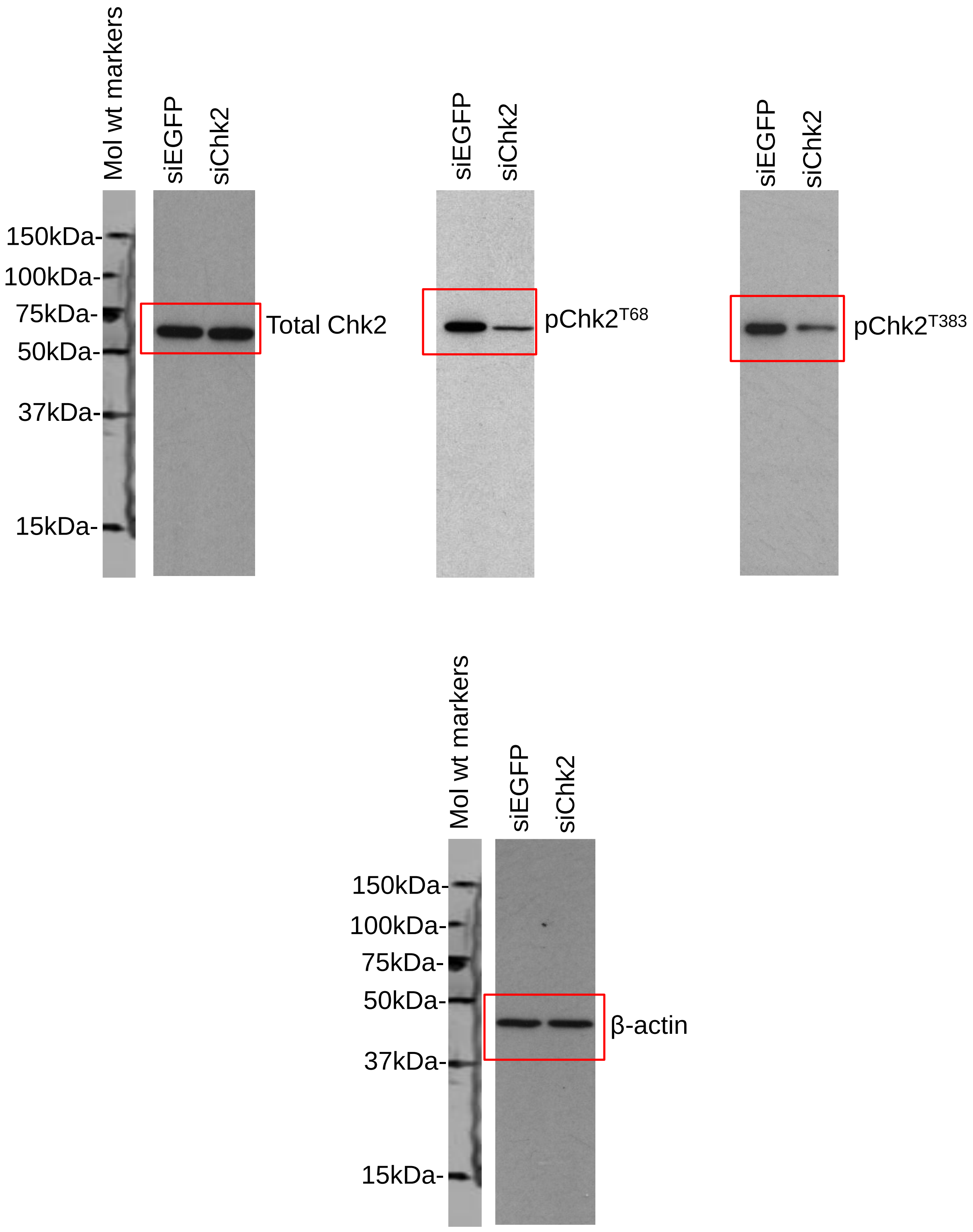

Fig. 3B Original blots

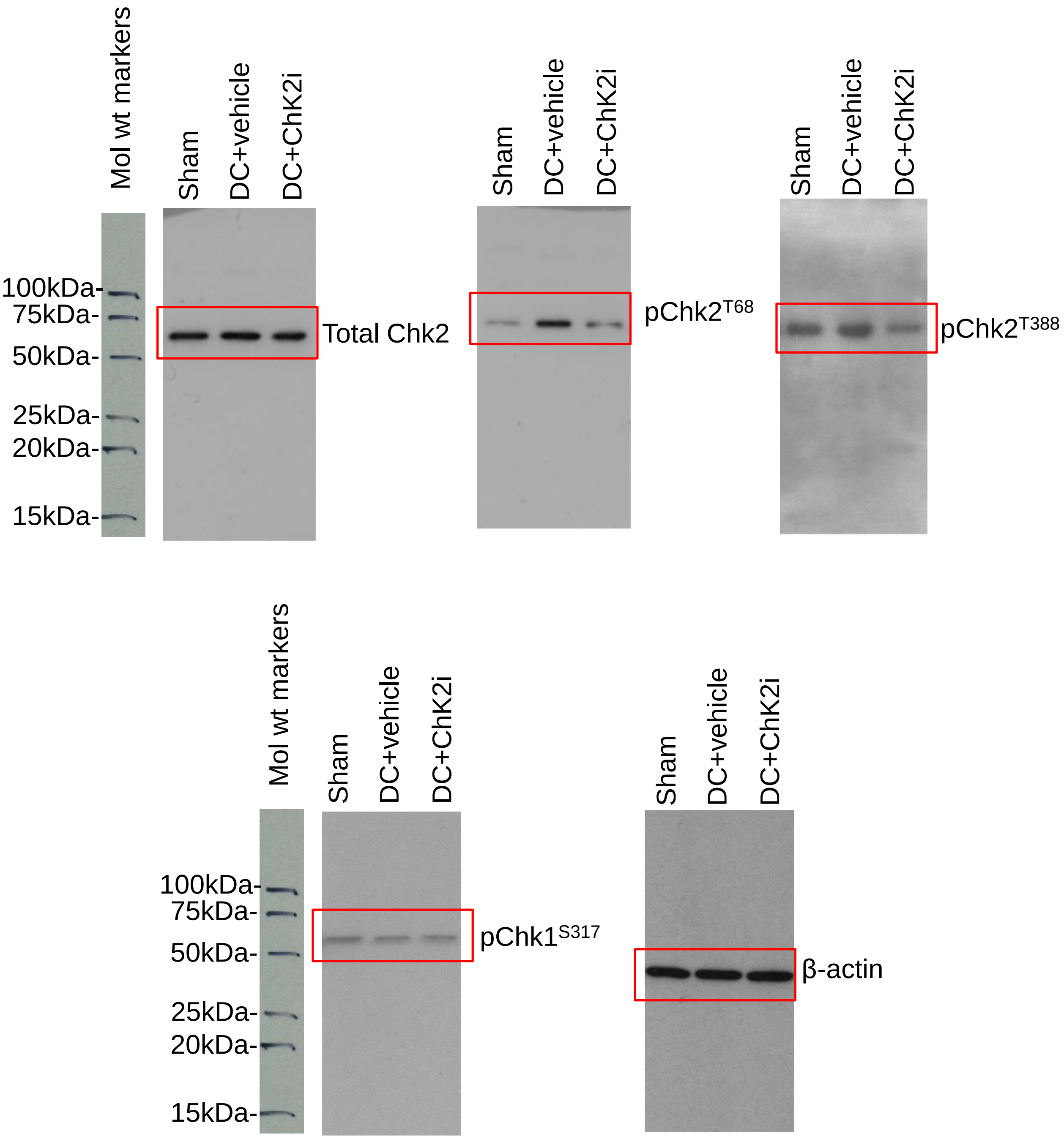

Fig. 4A Original blots

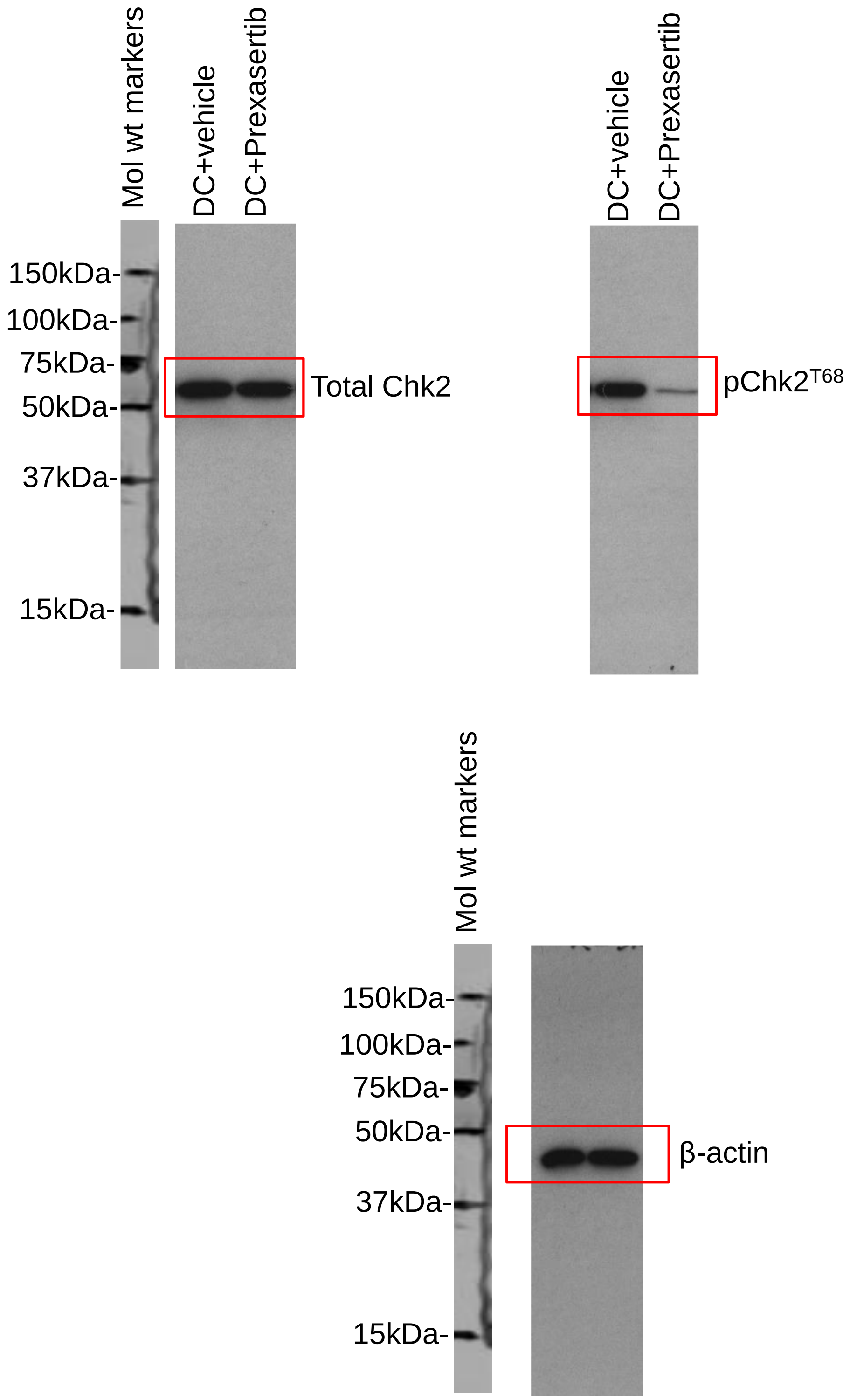

Fig. 5A original blots

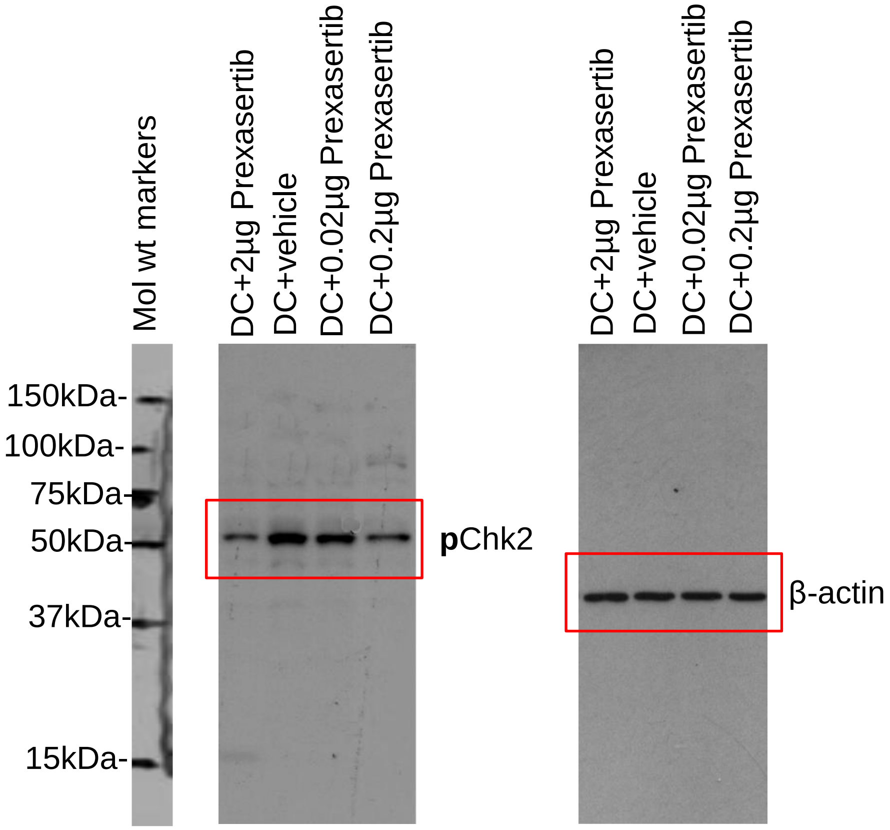

Fig. 7A original blots

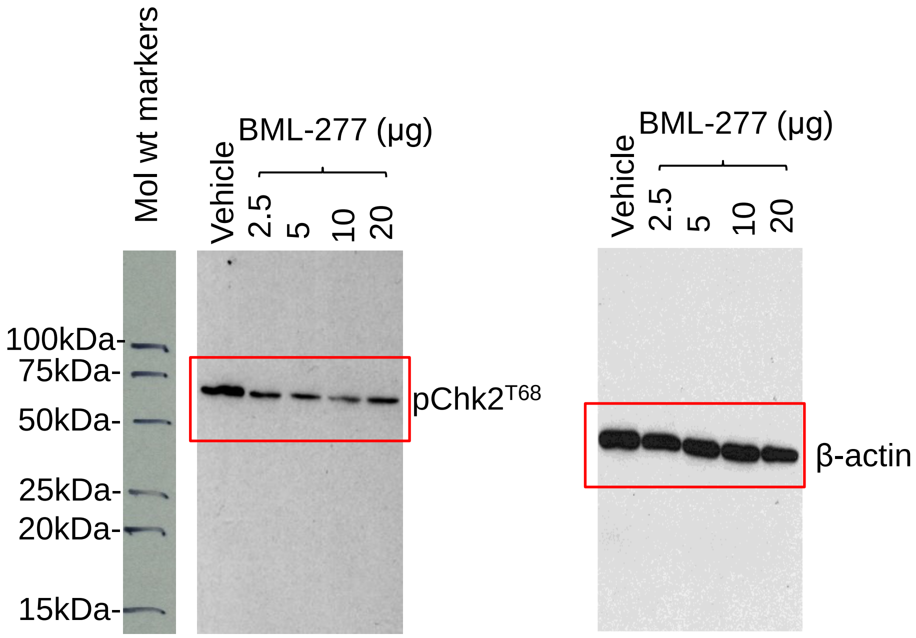

Fig. S4A original blots

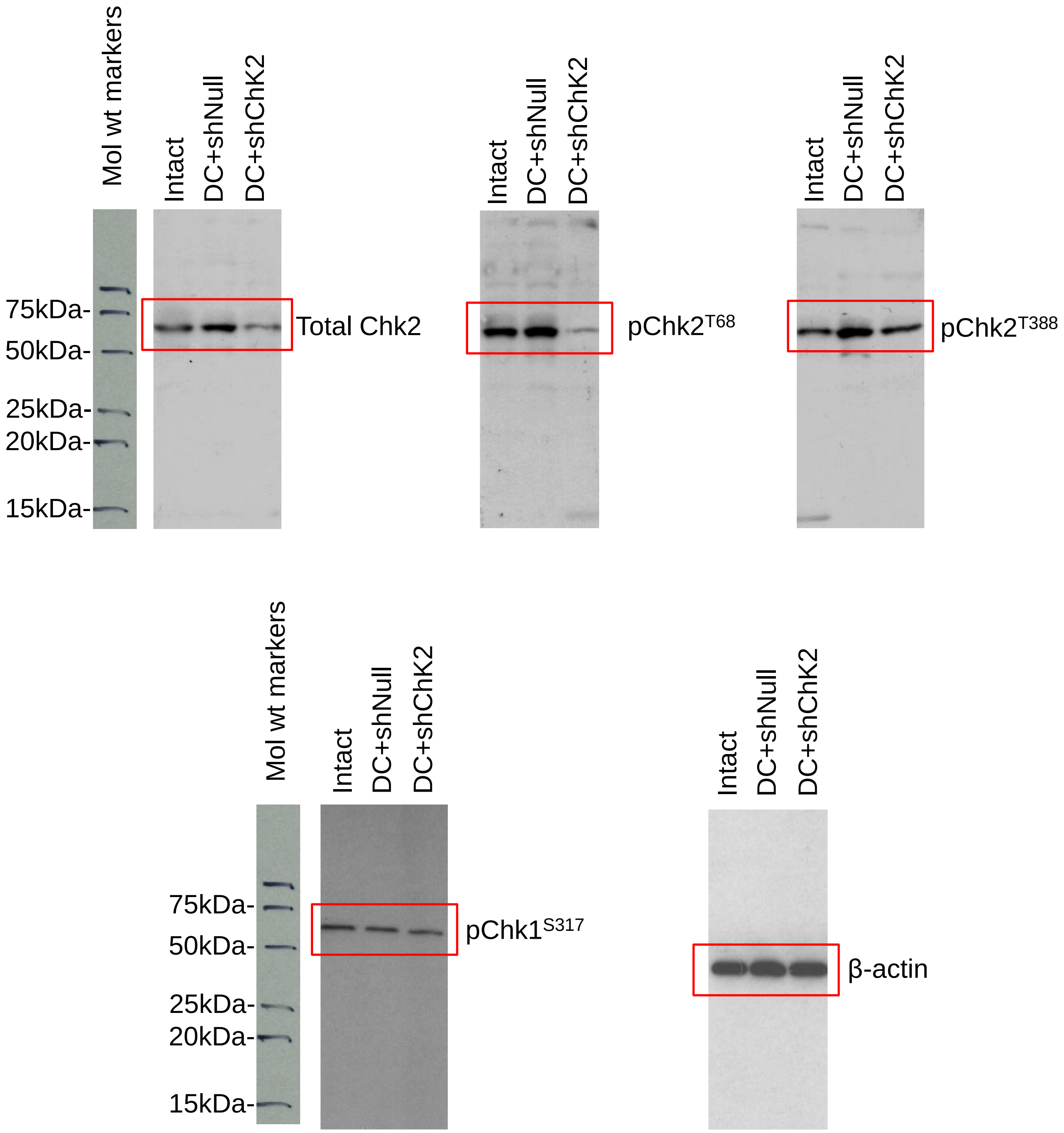

Fig. S5A original blots
